# Perioperative Events Following Open Versus Endovascular Revascularization for Chronic Limb-Threatening Ischemia: An NSQIP Analysis

**DOI:** 10.1016/j.jscai.2025.102579

**Published:** 2025-04-01

**Authors:** Waseem Wahood, Edwin A. Takahashi, Robert Lookstein, Eric A. Secemsky, Randall R. DeMartino, Joshua Beckman, Michael S. Conte, Sanjay Misra

**Affiliations:** a Department of Interventional Radiology, Jackson Memorial Hospital, University of Miami Miller School of Medicine, Miami, Florida; b Department of Radiology, Division of Vascular and Interventional Radiology, Mayo Clinic, Rochester, Minnesota; c Department of Diagnostic, Molecular, and Interventional Radiology, Icahn School of Medicine at Mount Sinai, New York, New York; d Division of Cardiology, Department of Medicine, Beth Israel Deaconess Medical Center, Boston, Massachusetts; e Department of Surgery, Division of Vascular and Endovascular Surgery, Mayo Clinic, Rochester, Minnesota; f Department of Medicine, University of Texas Southwestern, Dallas, Texas; g Division of Vascular and Endovascular Surgery, University of California, San Francisco, San Francisco, California

**Keywords:** BEST-CLI, chronic limb-threatening ischemia, endovascular, National Surgical Quality Improvement Project, surgical bypass

## Abstract

**Background:**

The Best Endovascular vs Best Surgical Therapy in Patients with Critical Limb Ischemia (BEST-CLI) trial reported the superiority of surgical bypass compared with endovascular intervention for the treatment of chronic limb-threatening ischemia (CLTI) in patients deemed suitable for either; however, the generalizability of these findings to the broader CLTI population is in question. Herein, we analyzed perioperative (30-day) outcomes from the National Surgical Quality Improvement Project (NSQIP) for CLTI interventions.

**Methods:**

The NSQIP-Vascular targeted database was queried from 2014 to 2019, contemporaneous with BEST-CLI, for patients undergoing CLTI intervention. Surgical bypass groups included saphenous vein (OPEN-GSV) or alternative conduit (OPEN-Other) and were compared to the endovascular (intervention group) (ENDO). Inverse Probability weighting with regression adjustment assessed 30-day outcomes including perioperative death (POD), major amputation, major adverse limb events (MALE, major reintervention and/or amputation), composite MALE or POD, and major adverse cardiovascular events (MACE, myocardial infarction, or stroke). Results were provided as risk ratio (RR).

**Results:**

Of the total cohort, 6780 (34.1%) were in the OPEN-GSV group, 4201 (21.1%) in OPEN-Other, and 8887 (44.7%) in ENDO. Compared to OPEN-GSV, ENDO exhibited a higher risk for major amputation (RR, 1.38; *P* = .002), higher risk of MALE (RR, 1.23; *P* = .004), lower risk of MACE (RR, 0.48; *P* < .001), and similar risk for all other outcomes. Compared to OPEN-Other, ENDO exhibited a lower risk of MACE (RR, 0.49; *P* < .001) and POD (RR, 0.76; *P* = .040) and was similar for all other outcomes.

**Conclusions:**

These data demonstrate a higher rate of early amputation and MALE among those who underwent ENDO vs OPEN-GSV. Conversely, early limb events were similar between ENDO and OPEN-Other. Both OPEN-GSV and OPEN-Other were associated with a higher risk of 30-day MACE. OPEN-Other was associated with a higher risk of MACE. These data highlight the importance of patient selection to optimize overall patient outcomes in CLTI.

## Introduction

Peripheral artery disease (PAD) affects approximately 230 million patients worldwide. Of these, roughly 11% present with chronic limb-threatening ischemia (CLTI), which has a poor limb and survival prognosis.^1^ The estimated annual cost of CLTI in the United States is approximately $12 billion.^2^

There are several guideline-targeted treatment options for CLTI, including medical management to lower cardiovascular and limb risk, endovascular revascularization with angioplasty and stenting, surgical bypass using synthetic or autogenous grafts, and local wound care to control infection.^3^ Without treatment, limb amputation or death are common late sequelae of this disease process.^4^^,^^5^ The choice of treatment varies among providers, severity of disease, and geographic regions.^6^, ^7^, ^8^ Anantha-Narayan et al^1^ demonstrated that endovascular therapy became increasingly more common compared to surgical bypass over the past decade.^1^ Yet, a recent randomized clinical trial by Farber et al^9^ shed some light on the debate on whether patients would benefit more from endovascular therapy or surgical bypass. In the Best Endovascular vs Best Surgical Therapy in Patients with Critical Limb Ischemia (BEST-CLI) trial, among patients with CLTI who had an adequate quality great saphenous vein and anatomy suitable for either surgical or endovascular revascularization, the incidence of a major adverse limb event (MALE) or death was lower for those who received surgical treatment than those treated with endovascular therapy.

As the BEST-CLI trial results are incorporated into the practice of peripheral vascular care, a comparison to real-world experience may further contextualize the trial data and facilitate identifying a suitable patient profile for each procedure. Herein, we analyzed the National Surgical Quality Improvement Project (NSQIP) for patients receiving intervention for CLTI with and without a viable saphenous vein for bypass and compared their outcomes to patients with CLTI who underwent endovascular therapy.

## Methods

### Data source

The NSQIP database was queried for our study. This database, created by the American College of Surgeons, collects a sample of deidentified data by trained abstractors from over 700 hospitals across the United States for preoperative and 30-day postoperative variables regarding randomly selected patients. These data are carefully reviewed by NSQIP representatives in participating hospitals; patients with incomplete 30-day follow-up are excluded from the database. Additionally, NSQIP contains procedure-targeted data and a vascular-targeted database, with more granular data points, including those specifically related to vascular and endovascular surgery. NSQIP’s vascular-targeted database was queried from 2014 to 2019 for patients who underwent intervention for CLTI, including tissue loss and rest pain. Because this is a deidentified patient database, the need for IRB approval and patient consent was waived. Patients with asymptomatic PAD, claudication, and acute limb ischemia were excluded. Like the BEST-CLI trial, the groups comprised: (1) surgical bypass patients with a suitable saphenous vein graft (OPEN-GSV); (2) those who received a bypass with prosthetic, spliced vein, or composite bypass graft (OPEN-Other); and (3) endovascular cohort was selected based on the primary procedure being angioplasty, stenting, or atherectomy (endovascular (intervention group) [ENDO]).

### Outcomes and covariates

Potential covariates in the database were queried. These included age, gender, race, body mass index (BMI), American Society of Anesthesiologists classification, functional status, operative time, inpatient/outpatient procedure, elective/emergency procedure, anatomical location of intervention, and comorbidities. Data on the anatomic complexity of disease treated, including the number of vessel runoff, tortuosity of vessels, and access complications, among others, are not available in this registry.

NSQIP-Vascular captures anatomical location differently between surgical and endovascular interventions. For the surgical groups, anatomical locations are separated by: femoral-popliteal, femoral-tibial/distal arteries (ie, distal to the popliteal artery), and popliteal-tibial/distal arteries. For the endovascular group, anatomical locations are separated by femoropopliteal and tibial.

Chronic limb-threatening ischemia-specific 30-day outcomes included major amputation (above-ankle), major reintervention (new surgical lower extremity bypass operation, surgical graft revision, bypass graft thrombectomy/thrombolysis), MALE (which includes major reintervention or major amputation), major adverse cardiovascular event (MACE, stroke, or myocardial infarction) and perioperative death (POD; within 30 days). CLTI intervention-specific variables were also included, such as symptomatology, ankle-brachial index, anatomic location, anatomic high-risk factors (prior intervention to index procedure, divided as femoral-popliteal level intervention or intervention distal to popliteal artery), physiologic high-risk factors (end-stage renal disease, congestive heart failure class III/IV, heart failure with reduced ejection fraction, etc), and preprocedural medications.

Secondary 30-day outcomes included discharge other than home (including against medical advice, hospice, senior community, rehab, acute care, skilled nursing facility, unskilled facility), wound infection, untreated loss of patency, bleeding requiring transfusions, the total length of stay, readmission, unplanned readmission, related readmission, serious complication (cardiac arrest, myocardial infarction, pneumonia, progressive renal insufficiency, acute renal failure, venous thromboembolism, return to the operating room, deep incisional surgical site infection (SSI), organ space SSI, systemic sepsis, unplanned intubation, urinary tract infection, wound disruption), and any complication (any SSI, wound disruption, pneumonia, unplanned intubation, PE, ventilator >48 hours, progressive renal insufficiency, acute renal failure, urinary tract infection, stroke, cardiac arrest, myocardial infarction, deep vein thrombosis, return to the operating room, systemic sepsis).

The NSQIP captures reasons for reoperation, readmission, and returning to the operating room; these variables were used to better understand whether the reason for these adverse events was related to the index procedure. NSQIP-Vascular also captures the “most severe outcome” which reports a hierarchical single most severe outcome; this variable was not used in the analysis but was recorded.

### Statistical analysis

Baseline categorical characteristics were represented by frequency and proportion and were compared using the χ^2^ test. Continuous variables were represented by mean and SD and were compared using the *t* test. OPEN-GSV and OPEN-Other were compared to ENDO, separately. Multivariable logistic regression was utilized to assess the association of procedure type with major amputation, MALE, POD, composite MALE or POD, MACE, and POD. Variables were selected based on univariate screening and clinical associations. These results were reported as odds ratios (OR) and their 95% CI. Additionally, inverse probability weighted regression adjustment (IPWRA) was used, in which the treatment multinomial logistic model was adjusted for anatomic and physiologic high-risk factors, anatomic location of treatment, age, gender, BMI, race, smoking status, and diabetes. The outcome logistic model was adjusted for age, gender, BMI, preprocedural medications, diabetes, smoking status, dyspnea, history of congestive heart failure, hypertension requiring medications, dialysis, disseminated cancer, weight loss, and bleeding disorder. These results were depicted as risk ratio (RR) and their corresponding 95% CI for approximate comparison of results with recent trials, which depict them as hazard ratios. A test of overidentification was conducted to assess that the treatment model balanced the covariates; *P* > .05 was the threshold for balance.^10^ The covariates were selected based on their corresponding univariate analysis and previous studies.^6^, ^7^, ^8^^,^^11^ IPWRA was selected as the statistical method as it is a causal inference methodology, addressing selection and confounding bias while providing more reliable estimates. The coefficients in this analysis are reported as average treatment effect, representing the difference in the mean expected effect of endovascular intervention compared to surgical intervention, averaged over all patients in the 2 groups.^12^^,^^13^ Although RCT are one of the strongest methods in establishing causal relationships, treatment assignment is not random in observational studies and introduces the risk of confounding bias. Therefore, the use of IPWRA attempts to address this issue by balancing measured covariates across procedure types. The regression adjustment component also controls the influence of covariates on the outcome. This dual approach provides a more robust causal effect estimate approximating the results of an RCT while acknowledging the limitations of a retrospective study.^13^ Due to the imbalance of groups regarding the anatomic location of intervention, IPWRA was conducted for femoral-popliteal, femoral-tibial, and popliteal-tibial interventions separately. The endovascular group is separated by femoropopliteal and tibial only; therefore, tibial/distal intervention in the ENDO group was used for comparison to the popliteal-tibial and femoral-tibial intervention in the OPEN groups. Additional IPWRA analysis was conducted, separated by symptomatology—rest pain and tissue loss. *P* < .05 was considered statistically significant. Statistical analysis was performed using STATA 17 (StataCorp LLC.).

## Results

### Patient characteristics

We identified 6780 patients in the OPEN-GSV group, 8887 in the ENDO group, and 4201 patients in the OPEN-Other group. The average age was 67.8 ± 11.6 years for the OPEN-GSV group, 69.6 ± 12.0 years for the ENDO group (OPEN-GSV vs ENDO: *P* < .001), and 69.4 ± 10.7 years in the OPEN-Other (vs ENDO: *P* = .27) group. The majority of procedures performed in the OPEN-GSV group involved the tibial/distal arteries (n = 3695, 54.5%), whereas more of the procedures in the ENDO group involved the femoral or popliteal arteries (n = 6118, 68.8%; OPEN-GSV vs ENDO: *P* < .001). The femoral and popliteal arteries were more commonly treated in the OPEN-Other group (n = 2434, 57.9%; vs 45.5% in the ENDO group: *P* < .001). There were statistically significant differences in functional health status (*P* < .001), and comorbidities such as dialysis (*P* < .001), bleeding disorder (*P* < .001), as well as anatomical location of intervention (*P* < .001), among others. More data on patient characteristics can be seen in Table 1, Table 2, Table 3.

**Table 1:** Patient demographics.

Variables	OPEN-GSV (n = 6780)	ENDO (n = 8887)	OPEN-Other (n = 4201)	*P* value (OPEN-GSV vs ENDO)	*P* value (OPEN-Other vs ENDO)
Age, y	67.81 ± 11.55	69.6 ± 11.96	69.36 ± 10.71	<.001	.268
BMI, kg/m^2^	27.91 ± 6.025	28.19 ± 6.44	27.16 ± 5.981	.007	<.001
Gender	2191 (32.32%)	3661 (41.20%)	1592 (37.90%)	<.001	<.001
Race				<.001	<.001
White	3886 (75.50%)	5499 (71.60%)	2536 (72.60%)		
Black or African American	1185 (23.02%)	1953 (25.43%)	908 (25.99%)		
Asian	54 (1.05%)	166 (2.16%)	33 (0.94%)		
American Indian or Alaska Native	18 (0.35%)	45 (0.59%)	13 (0.37%)		
Native Hawaiian or Pacific Islander	4 (0.08%)	17 (0.22%)	3 (0.09%)		
Functional health status				<.001	<.001
Independent	6222 (92.14%)	7426 (84.04%)	3719 (89.08%)		
Partially dependent	497 (7.36%)	1257 (14.23%)	410 (9.82%)		
Totally dependent	34 (0.50%)	153 (1.73%)	46 (1.10%)		
Diabetes				<.001	<.001
None	3334 (49.17%)	3424 (38.53%)	2050 (48.80%)		
Noninsulin dependent	1350 (19.91%)	1742 (19.60%)	816 (19.42%)		
Insulin-dependent	2096 (30.91%)	3721 (41.87%)	1335 (31.78%)		
Current smoker within 1 year	2830 (41.74%)	2392 (26.92%)	1629 (38.78%)	<.001	<.001
Dyspnea				<.001	.716
No	6129 (90.40%)	7,877 (88.64%)	3741 (89.05%)		
Moderate exertion	622 (9.17%)	933 (10.50%)	422 (10.05%)		
At rest	29 (0.43%)	77 (0.87%)	38 (0.90%)		
Ventilator dependent	7 (0.10%)	13 (0.15%)	7 (0.17%)	.455	.781
History of severe COPD	745 (10.99%)	899 (10.12%)	566 (13.47%)	.078	<.001
Ascites	9 (0.13%)	18 (0.20%)	5 (0.12%)	.297	.287
CHF with prior 30 days	207 (3.05%)	436 (4.91%)	189 (4.50%)	<.001	.308
Hypertension requiring medications	5417 (79.90%)	7535 (84.79%)	3579 (85.19%)	<.001	.543
Acute renal failure (preoperative)	85 (1.25%)	155 (1.74%)	64 (1.52%)	.013	.358
Currently on dialysis	484 (7.14%)	1248 (14.04%)	372 (8.86%)	<.001	<.001
Disseminated cancer	31 (0.46%)	49 (0.55%)	30 (0.71%)	.413	.262
Open wound/wound infection	3356 (49.50%)	4604 (51.81%)	1921 (45.73%)	.004	<.001
Steroid use for chronic condition	353 (5.21%)	616 (6.93%)	234 (5.57%)	<.001	.003
>10% loss of body weight in last 6 months	64 (0.94%)	125 (1.41%)	67 (1.59%)	.009	.403
Bleeding disorder	1280 (22.20%)	2351 (32.98%)	960 (26.67%)	<.001	<.001
Preop transfusion with 72 h prior to surgery	135 (1.99%)	151 (1.70%)	106 (2.52%)	.176	.002
Systemic sepsis				<.001	<.001
None	6449 (95.12%)	8213 (92.42%)	3986 (94.88%)		
Systemic inflammatory response syndrome	239 (3.53%)	503 (5.66%)	174 (4.14%)		
Sepsis	85 (1.25%)	163 (1.83%)	40 (0.95%)		
Septic shock	7 (0.10%)	8 (0.09%)	1 (0.02%)		

Values are mean ± SD or n (%).

BMI, body mass index; CHF, congestive heart failure; COPD, Chronic Obstructive Pulmonary Disease; ENDO, endovascular (intervention group).

**Table 2:** Procedural data.

Variables	OPEN-GSV (n = 6780)	ENDO (n = 8887)	OPEN-Other (n = 4201)	*P* value (OPEN-GSV vs ENDO)	*P* value (OPEN-Other vs ENDO)
Symptomatology				<.001	<.001
Critical limb ischemia: Rest pain	2611 (38.51%)	2709 (30.48%)	1858 (44.23%)		
Critical limb ischemia: Tissue loss	4169 (61.49%)	6178 (69.52%)	2343 (55.77%)		
High-risk factors, physiologic	1648 (24.48%)	3268 (36.95%)	1205 (28.8%)	<.001	<.001
High-risk factors, anatomic				.048	<.001
None	4362 (64.34%)	5843 (65.75%)	2129 (50.68%)		
Prior bypass	1103 (16.27%)	1321 (14.86%)	1316 (31.33%)		
Prior endovascular	1315 (19.40%)	1723 (19.39%)	756 (18.00%)		
Preprocedural antiplatelet medication	5333 (79.00%)	7226 (81.62%)	3513 (83.92%)	<.001	.001
Preprocedural medication-statin	4740 (70.25%)	6300 (71.23%)	3157 (75.47%)	.185	<.001
Preprocedural medication-beta blocker	3719 (55.17%)	5443 (61.57%)	2592 (61.95%)	<.001	.679
Overall procedure				<.001	<.001
Angioplasty	0 (0%)	4260 (47.94%)	0 (0%)		
Atherectomy	0 (0%)	1871 (21.05%)	0 (0%)		
Bypass	6780 (100%)	0 (0%)	4201 (100%)		
Stenting	0 (0%)	2756 (31.01%)	0 (0%)		
Anatomic location				<.001	<.001
Femoral/popliteal	3085 (45.50%)	6118 (68.84%)	2434 (57.94%)		
Tibial/distal	3695 (54.50%)	2769 (31.16%)	1767 (42.06%)		
Procedure				<.001	<.001
Femoral distal bypass	2658 (39.20%)	–	1498 (35.66%)		
Femoropopliteal angioplasty/stenting/atherectomy	–	6118 (68.84%)	–		
Femoropopliteal bypass	3085 (45.50%)	0 (0%)	2434 (57.94%)		
Popliteal distal	1037 (15.29%)	0 (0%)	269 (6.40%)		
Tibial angioplasty/stenting	–	2769 (31.16%)	–		
ASA class				<.001	<.001
1. No disturbance	6 (0.09%)	13 (0.15%)	2 (0.05%)		
2. Mild disturbance	201 (2.97%)	478 (5.69%)	72 (1.72%)		
3. Severe disturbance	4623 (68.30%)	5464 (64.99%)	2757 (65.71%)		
4. Life threatening	1928 (28.48%)	2442 (29.04%)	1362 (32.46%)		
5. Moribund	11 (0.16%)	11 (0.13%)	3 (0.07%)		
Anesthesia type				<.001	<.001
General	6191 (91.34%)	3195 (35.96%)	4011 (95.50%)		
MAC/IV sedation	151 (2.23%)	5448 (61.32%)	57 (1.36%)		
Regional	22 (0.32%)	43 (0.48%)	10 (0.24%)		
Epidural	46 (0.68%)	1 (0.01%)	1 (0.02%)		
Spinal	356 (5.25%)	32 (0.36%)	6 (0.14%)		
None	0 (0%)	35 (0.39%)	17 (0.40%)		
Other	12 (0.18%)	18 (0.20%)	98 (2.33%)		
Local	0 (0%)	112 (1.26%)	0 (0%)		
Surgical specialties				<.001	<.001
Interventional radiologist	0 (0%)	176 (1.98%)	0 (0%)		
Vascular	6658 (98.20%)	8569 (96.42%)	4122 (98.12%)		
Other	122 (1.82%)	142 (1.60%)	79 (1.88%)		
Elective surgery	3593 (53.06%)	4397 (49.49%)	2168 (51.63%)	<.001	.022
Operative time	254.2 ± 109.5	112.8 ± 71.81	229.3 ± 113.7	<.001	<.001

Values are n (%) or mean ± SD.

ASA, American Society of Anesthesiologists; ENDO, endovascular (intervention group); MAC/IV, monitored anesthesia care/intravenous.

**Table 3:** Thirty-day outcomes.

Variables	OPEN-GSV (n = 6780)	ENDO (n = 8887)	OPEN-Other (n = 4201)	*P* value (OPEN-GSV vs ENDO)	*P* value (OPEN-Other vs ENDO)
Total length of stay, d	9.755 ± 8.538	5.964 ± 8.314	9.549 ± 8.333	<.001	<.001
Discharge disposition				<.001	<.001
Home	4337 (64.48%)	7018 (79.34%)	2546 (60.84%)		
AMA	14 (0.21%)	19 (0.21%)	6 (0.14%)		
Expired	87 (1.29%)	92 (1.04%)	79 (1.89%)		
Hospice	8 (0.12%)	31 (0.35%)	5 (0.12%)		
Multilevel senior community	2 (0.03%)	0 (0.00%)	2 (0.05%)		
Rehab	858 (12.76%)	533 (6.03%)	565 (13.50%)		
Separate acute care	126 (1.87%)	83 (0.94%)	69 (1.65%)		
Skilled care, not home	1274 (18.94%)	1053 (11.90%)	897 (21.43%)		
Unskilled facility, not home	20 (0.30%)	17 (0.19%)	16 (0.38%)		
Discharge other than home	2356 (35.20%)	1777 (20.20%)	1576 (38.23%)	<.001	<.001
Untreated loss of patency	137 (2.02%)	149 (1.68%)	91 (2.17%)	.111	.051
Bleeding requiring transfusion or secondary procedure	1241 (18.30%)	709 (7.98%)	913 (21.73%)	<.001	<.001
MACE	266 (3.92%)	194 (2.18%)	165 (3.93%)	<.001	<.001
Wound infection/complication	988 (14.57%)	224 (2.52%)	556 (13.23%)	<.001	<.001
Major reintervention of the treated segment	347 (5.12%)	395 (4.44%)	233 (5.55%)	.049	.006
Major amputation (transtibial or proximal)	235 (3.47%)	427 (4.80%)	207 (4.93%)	<.001	.76
MALE	524 (7.73%)	768 (8.64%)	379 (9.02%)	.04	.473
POD	159 (2.35%)	244 (2.75%)	124 (2.95%)	.117	.505
MALE or POD	656 (9.68%)	973 (10.95%)	480 (11.40%)	.01	.417
Readmission	1184 (19.96%)	1490 (20.06%)	771 (20.84%)	.884	.336
Unplanned readmission	1134 (19.28%)	1358 (18.62%)	740 (20.17%)	.333	.051
Related readmission	883 (15.68%)	768 (11.46%)	577 (16.46%)	<.001	<.001
Serious complication	1795 (26.47%)	1690 (19.02%)	1142 (27.18%)	<.001	<.001
Any complication	2096 (30.91%)	1745 (19.64%)	1270 (30.23%)	<.001	<.001
Cardiac arrest	78 (1.15%)	74 (0.83%)	58 (1.38%)	.044	.003
Myocardial infarction	253 (3.73%)	192 (2.16%)	161 (3.83%)	<.001	<.001
Cardiac complication	309 (4.56%)	245 (2.76%)	207 (4.93%)	<.001	<.001
Pneumonia	113 (1.67%)	132 (1.49%)	68 (1.62%)	.365	.562
Deep incisional SSI	159 (2.35%)	35 (0.39%)	95 (2.26%)	<.001	<.001
Organ space SSI	49 (0.72%)	28 (0.32%)	58 (1.38%)	<.001	<.001
Superficial incisional SSI	499 (7.36%)	72 (0.81%)	216 (5.14%)	<.001	<.001
SSI	695 (10.25%)	135 (1.52%)	361 (8.59%)	<.001	<.001
Urinary tract infection	97 (1.43%)	73 (0.82%)	86 (2.05%)	<.001	<.001
Venous thromboembolism	73 (1.08%)	62 (0.70%)	38 (0.90%)	.011	.204
Acute renal failure	38 (0.56%)	54 (0.61%)	20 (0.48%)	.702	.349
Progressive renal insufficiency	41 (0.60%)	47 (0.53%)	23 (0.55%)	.529	.892
Renal Failure	78 (1.15%)	100 (1.13%)	43 (1.02%)	.883	.601
Return to OR	1219 (17.98%)	1176 (13.23%)	765 (18.21%)	<.001	<.001
Reoperation	1220 (17.99%)	1176 (13.23%)	765 (18.21%)	<.001	<.001
Wound disruption	112 (1.65%)	24 (0.27%)	73 (1.74%)	<.001	<0.001
Deep vein thrombosis	55 (0.81%)	51 (0.57%)	34 (0.81%)	.073	.117
Pulmonary embolism	22 (0.32%)	13 (0.15%)	5 (0.12%)	.019	.694
Unplanned intubation	123 (1.81%)	126 (1.42%)	78 (1.86%)	.049	.058
Failure to wean	66 (0.97%)	68 (0.77%)	42 (1.00%)	.161	.17
Stroke	42 (0.62%)	38 (0.43%)	28 (0.67%)	.095	.072
Septic shock	75 (1.11%)	66 (0.74%)	48 (1.14%)	.017	.022
Sepsis	145 (2.14%)	188 (2.12%)	112 (2.67%)	.921	.049

Values are n (%) or mean ± SD.

AMA, against medical advice; CVA, cerebrovascular accident; ENDO, endovascular (intervention group); MACE, major adverse cardiovascular event; MALE, major adverse limb event; OR, operating room; POD, perioperative death; SSI, surgical site infection.

### Multivariable regression

Compared to the ENDO group, the OPEN-GSV group was associated with lower rates of major amputations (OR, 0.74; 95% CI, 0.61-0.90; *P* = .002) and MALE (OR, 0.84; 95% CI, 0.73-0.96; *P* = .013). There was no significant difference between the OPEN-GSV and ENDO groups for the outcomes of POD (OR, 1.21; 95% CI, 0.96-1.53; *P* = .11), composite MALE or POD (OR, 0.91; 95% CI, 0.80-1.03; *P* = .14), and major reintervention (OR, 1.01; 95% CI, 0.84-1.20; *P* = .93). OPEN-GSV was associated with higher odds of MACE compared to the ENDO group (OR, 2.07; 95% CI, 1.60-2.69; *P* < .001).

Compared to the ENDO group, the OPEN-Other group was associated with similar rates of major amputation (OR, 0.99; 95% CI, 0.81-1.22; *P* = .95), MALE (OR, 0.97; 95% CI, 0.84-1.13; *P* = .72), composite MALE or POD (OR, 1.03; 95% CI, 0.90-1.18; *P* = .68), and major reintervention (OR,1.10; 95% CI, 0.91-1.33; *P* = .32). Additionally, the OPEN-Other group was associated with higher odds of POD (OR, 1.37; 95% CI, 1.07-1.75; *P* = .012) and MACE (OR, 2.15; 95% CI, 1.64-2.81; *P* < .001) compared to the ENDO group.

Prior bypass to index procedure was associated with higher odds of major amputation (OR, 1.79; 95% CI, 1.47-2.17; *P* < .001), MALE (OR,1.85; 95% CI, 1.60-2.13; *P* < .001), composite MALE or POD (OR, 1.62; 95% CI, 1.42-1.85; *P* < .001), and major reintervention (OR, 1.89; 95% CI, 1.59-2.26; *P* < .001). Additionally, the prior bypass was associated with similar odds of POD (OR, 0.82; 95% CI, 0.62-1.10; *P* = .19) and MACE (OR, 0.95; 95% CI, 0.72-1.27; *P* = .74).

Prior endovascular intervention to index procedure was associated with higher odds of MALE (OR, 1.19; 95% CI, 1.02-1.39; *P* = .025), composite MALE or POD (OR, 1.17; 95% CI, 1.02-1.34; *P* = .028), and major reintervention (OR, 1.36; 95% CI, 1.12-1.64; *P* = .002). Additionally, prior endovascular reintervention was associated with similar odds of major amputation (OR, 0.98; 95% CI, 0.79-1.22; *P* = .87), POD (OR, 1.06; 95% CI, 0.83-1.35; *P* = .65), and MACE (OR, 1.04; 95% CI, 0.80-1.36; *P* = .76).

Male patients had similar odds of major amputation (OR, 1.04; 95% CI, 0.88-1.23; *P* = .64), MALE (OR, 0.98; 95% CI, 0.87-1.10; *P* = .71), POD (OR, 0.98; 95% CI, 0.80-1.19; *P* = .81), composite MALE or POD (OR, 0.96; 95% CI, 0.86-1.07; *P* = .48), major reintervention (OR, 0.91; 95% CI, 0.78-1.06; *P* = .22), and MACE (OR, 0.96; 95% CI, 0.77-1.20; *P* = .72) compared to female patients.

Compared to White patients, Black patients were associated with higher odds of major amputation (OR, 1.35; 95% CI, 1.14-1.60; *P* < .001), MALE (OR, 1.14; 95% CI, 1.00-1.30; *P* = .048). They had similar odds of composite MALE or POD (OR, 1.03; 95% CI, 0.91-1.17; *P* = .61), major reintervention (OR, 0.96; 95% CI, 0.81-1.14; *P* = .64), and MACE (OR, 0.78; 95% CI, 0.60-1.01; *P* = .058). Race was not significant in the univariate analysis for POD.

These results can be seen in Supplemental Table S1.

### Inverse probability weighting with regression adjustment analysis

Compared to the OPEN-GSV group, the ENDO group was associated with a higher risk of major amputation (RR, 1.38; 95% CI, 1.12-1.71; *P* = .002), higher risk of MALE (RR, 1.23; 95% CI, 1.06-1.42; *P* = .005), similar risk of MALE or POD (RR, 1.11; 95% CI, 0.98-1.26; *P* = .11), similar risk of mortality (RR, 0.78; 95% CI, 0.60-1.01; *P* = .058), similar risk of major reintervention (RR, 1.02; 95% CI, 0.85-1.24; *P* = .81), and lower risk of MACE (RR, 0.48; 95% CI, 0.37-0.62; *P* < .001).

Compared to the OPEN-Other group, the ENDO group was associated with a similar risk of major amputation (RR, 1.03; 95% CI, 0.84-1.27; *P* = .76), similar risk of MALE (RR, 1.07; 95% CI, 0.93-1.24; *P* = .35), similar risk of MALE or POD (RR, 1.01; 95% CI, 0.88-1.14; *P* = .93), lower risk of POD (RR, 0.76; 95% CI, 0.59-0.99; *P* = .040), similar risk of major reintervention (RR, 0.93; 95% CI, 0.77-1.14; *P* = .49), and lower risk of MACE (RR, 0.49; 95% CI, 0.37-0.63; *P* < .001).

**Figure 1:**
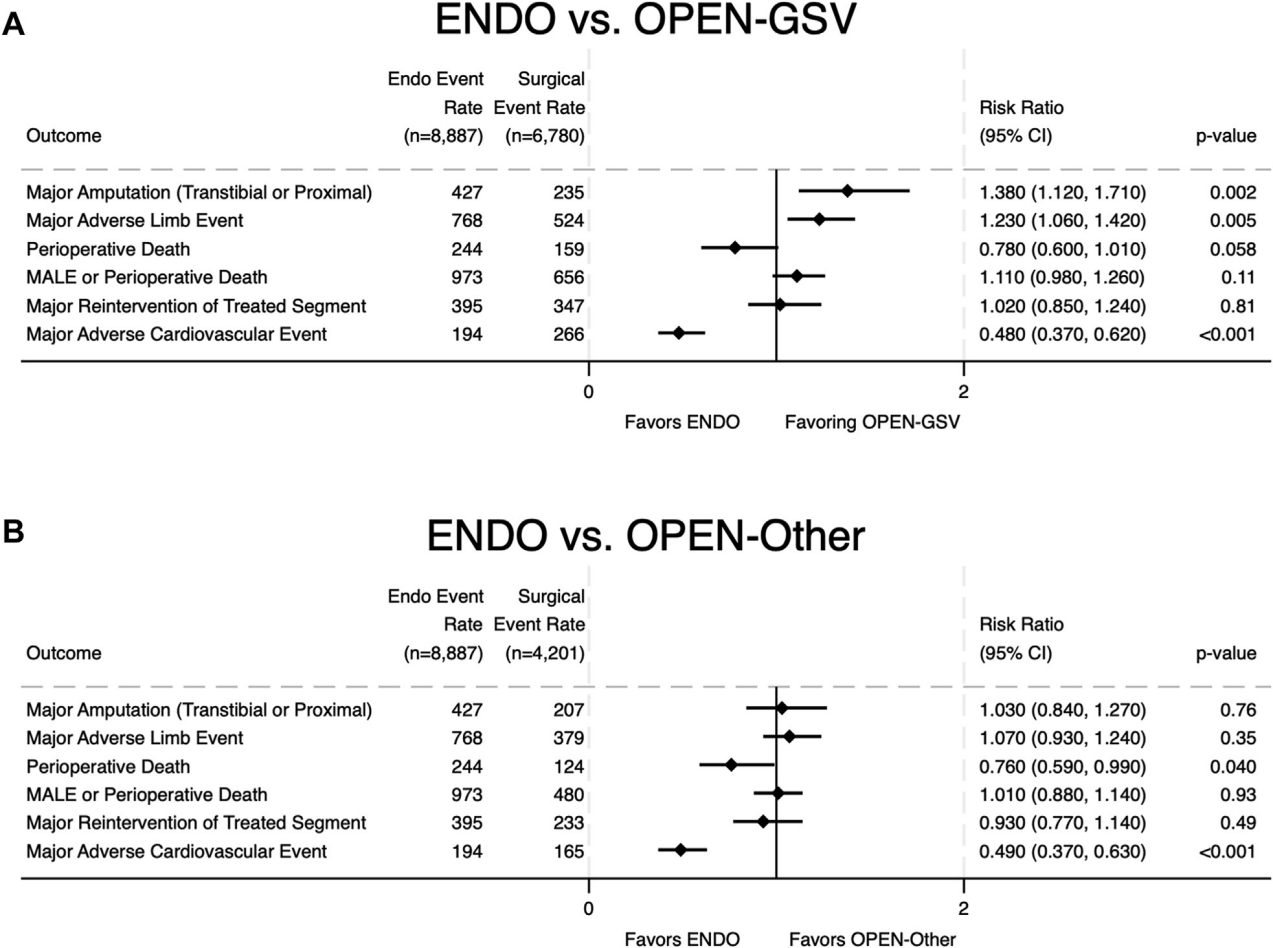
**Regression analysis of 30-day outcomes for (A) the ENDO vs OPEN-GSV and (B) ENDO vs OPEN-Other groups.** ENDO, endovascular (intervention group); MALE, major adverse limb event.

Results from these analyses are displayed in Table 4 and Figure 1.

**Table 4:** Inverse-propensity weighted with regression adjustment analysis.

Variable	Risk ratio	95% CI	*P* value
ENDO vs OPEN-GSV			
Major amputation	1.38	1.12-1.71	.002
MALE	1.23	1.06-1.42	.005
POD	0.78	0.60-1.01	.058
MALE or POD	1.11	0.98-1.26	.11
Major reintervention	1.02	0.85-1.24	.81
MACE	0.48	0.37-0.62	<.001

ENDO vs OPEN-Other			

Major amputation	1.03	0.84-1.27	.76
MALE	1.07	0.93-1.24	.35
POD	0.76	0.59-0.99	.040
MALE or POD	1.01	0.88-1.14	.93
Major reintervention	0.93	0.77-1.14	.49
MACE	0.49	0.37-0.63	<.001

MACE, major adverse cardiovascular event; MALE, major adverse limb event; POD, perioperative death.

### Inverse probability weighting with regression adjustment: Rest pain

Compared to the OPEN-GSV group, the ENDO group was associated with a higher risk of major amputation (RR, 1.67; 95% CI, 1.14-2.46; *P* = .009), higher risk of MALE (RR, 1.45; 95% CI, 1.15-1.84; *P* = .002), and higher risk of MALE or POD (RR, 1.32; 95% CI, 1.06-1.64; *P* = .012). The ENDO group was associated with similar risk for mortality (RR, 0.88; 95% CI, 0.55-1.42; *P* = .60), similar risk for major reintervention (RR, 1.33; 95% CI, 1.00-1.77; *P* = .051), and similar risk for MACE (RR, 0.75; 95% CI, 0.47-1.19; *P* = .21).

Compared to the OPEN-Other group, the ENDO group was associated with a similar risk of major amputation (RR, 1.24; 95% CI, 0.87-1.75; *P* = .23), similar risk of MALE (RR, 1.25; 95% CI, 0.99-1.57; *P* = .055), similar risk of MALE or POD (RR, 1.16; 95% CI, 0.95-1.42; *P* = .15), similar risk of mortality (RR, 0.92; 95% CI, 0.58-1.44; *P* = .71), similar risk of major reintervention (RR, 1.31; 95% CI, 0.98-1.74; *P* = .07), and similar risk of MACE (RR, 0.66; 95% CI, 0.43-1.01; *P* = .055).

These results may be seen in Supplemental Table S2.

### Inverse probability weighting with regression adjustment: Tissue loss

Compared to the OPEN-GSV group, the ENDO group was associated with a similar risk of major amputation (RR, 1.27; 95% CI, 0.99-1.63; *P* = .06), similar risk of MALE (RR, 1.15; 95% CI, 0.95-1.38; *P* = .14), a similar risk of MALE or POD (RR, 1.02; 95% CI, 0.88-1.20; *P* = .77), lower risk of mortality (RR, 0.71; 95% CI, 0.53-0.97; *P* = .028), similar risk of major reintervention (RR, 0.88; 95% CI, 0.68-1.13; *P* = .30), and lower risk of MACE (RR, 0.39; 95% CI, 0.29-0.53; *P* < .001).

Compared to the OPEN-Other group, the ENDO group was associated with a similar risk of major amputation (RR, 1.10; 95% CI, 0.86-1.39; *P* = .46), similar risk of MALE (RR, 1.04; 95% CI, 0.87-1.25; *P* = .65), similar risk of MALE or POD (RR, 0.94; 95% CI, 0.81-1.10; *P* = .46), lower risk of mortality (RR, 0.64; 95% CI, 0.48-0.86; *P* = .003), similar risk of major reintervention (RR, 0.79; 95% CI, 0.61-1.01; *P* = .06), and lower risk of MACE (RR, 0.44; 95% CI, 0.32-0.59; *P* < .001). These results may be seen in Supplemental Table S2.

### Patient characteristics: Femoral-popliteal

We identified a total of 3085 patients in the OPEN-GSV group, 6118 in the ENDO group, and 2434 patients in the OPEN-Other group. The average age was 67.2 ± 11.1 years for the OPEN-GSV group, 69.7 ± 11.8 years for the ENDO group (OPEN-GSV vs ENDO: *P* < .001), and 69.0 ± 10.7 years in the OPEN-Other group (vs ENDO: *P* = .009). There was a higher proportion of male patients compared to female patients in all 3 groups (OPEN-GSV vs ENDO: 62.9% vs 54.7%; *P* < .001; OPEN-Other vs ENDO: 59.6% vs 54.7%; *P* < .001). More data on patient characteristics can be seen in Supplemental Tables S3-S5.

### Multivariable regression: Femoral-popliteal

Compared to the ENDO group, the OPEN-GSV group was associated with lower rates of major amputations (OR, 0.59; 95% CI, 0.43-0.80; *P* = .001), MALE (OR, 0.71; 95% CI, 0.58-0.87; *P* = .001), and composite MALE or POD (OR, 0.82; 95% CI, 0.68-0.99; *P* = .035), There was no significant difference between the OPEN-GSV and ENDO groups for the outcomes of POD (OR, 1.25; 95% CI, 0.90-1.73; *P* = .18), and major reintervention (OR, 0.86; 95% CI, 0.67-1.11; *P* = .25). The OPEN-GSV group was associated with higher odds of MACE compared to the ENDO group (OR 1.66; 95% CI, 1.17-2.35; *P* = .004).

Compared to the ENDO group, the OPEN-Other group was associated with similar rates of major amputation (OR, 0.77; 95% CI, 0.58-1.02; *P* = .069) and POD (OR, 1.24; 95% CI, 0.91-1.70; *P* = .17). The OPEN-Other group was associated with lower odds of MALE (OR, 0.69; 95% CI, 0.56-0.85; *P* = .001), composite MALE or POD (OR, 0.78; 95% CI, 0.65-0.94; *P* = .008), and major reintervention (OR, 0.76; 95% CI, 0.59-0.99; *P* = .046). Additionally, the OPEN-Other group was associated with higher odds of MACE (OR, 1.63; 95% CI, 1.16-2.29; *P* = .005) compared to the ENDO group. Additional results may be seen in Supplemental Table S6.

### Sensitivity analysis: IPWRA—Femoral-popliteal

Compared to the OPEN-GSV group, the ENDO group was associated with a higher risk of major amputation (RR, 1.50; 95% CI, 1.07-2.10; *P* = .019), higher risk of MALE (RR, 1.33; 95% CI, 1.07-1.65; *P* = .010), and similar risk of MALE or POD (RR, 1.16; 95% CI, 0.97-1.40; *P* = .11), similar risk of mortality (RR, 0.77; 95% CI, 0.53-1.12; *P* = .17), similar risk of major reintervention (RR, 1.14; 95% CI, 0.87-1.50; *P* = .34), and lower risk of MACE (RR, 0.64; 95% CI, 0.46-0.90; *P* = .011).

Compared to the OPEN-Other group, the ENDO group was associated with similar risk of major amputation (RR, 1.29; 95% CI, 0.96-1.74; *P* = .09), higher risk of MALE (RR, 1.45; 95% CI, 1.17-1.79; *P* < .001), higher risk of MALE or POD (RR, 1.26; 95% CI, 1.06-1.51; *P* = .011), similar risk of mortality (RR, 0.79; 95% CI, 0.57-1.09; *P* = .15), similar risk of major reintervention (RR, 1.31; 95% CI, 0.99-1.75; *P* = .059), and lower risk of MACE (RR, 0.60; 95% CI, 0.43-0.84; *P* = .003).

Results from these analyses are displayed in Supplemental Table S7.

### Patient characteristics: Femoral-tibial

We identified a total of 2769 patients in the ENDO group, 2658 in the OPEN-GSV group, and 1498 patients in the OPEN-Other group. The average age was 69.4 ± 12.2 years for the ENDO group, 68.5 ± 11.8 years for the OPEN-GSV group (ENDO vs OPEN-GSV: *P* = .006), and 70.0 ± 10.8 years in the OPEN-Other group (vs ENDO: *P* = .08). There was a higher proportion of male patients compared to female patients in all 3 groups (ENDO vs OPEN-GSV: 68% vs 71%; *P* = .009; ENDO vs OPEN-Other: 68% vs 71%; *P* = .056). More data on patient characteristics can be seen in Supplemental Tables S8-S10.

### Multivariable regression: Femoral-tibial

Compared to the ENDO group, the OPEN-GSV group was associated with similar rates of major amputations (OR, 1.04; 95% CI, 0.77-1.42; *P* = .780), MALE (OR, 1.21; 95% CI, 0.96-1.51; *P* = .108), POD (OR, 1.17; 95% CI, 0.78-1.75; *P* = .44), composite MALE or POD (OR, 1.15; 95% CI, 0.94-1.42; *P* = .18). The OPEN-GSV group was associated with higher odds of major reintervention (OR, 1.46; 95% CI, 1.08-1.96; *P* = .013) and MACE compared to the ENDO group (OR 2.99; 95% CI, 1.85-4.83; *P* < .001).

Compared to the ENDO group, the OPEN-Other group was associated with higher rates of major amputation (OR, 1.62; 95% CI, 1.18-2.24; *P* = .003), MALE (OR, 1.69; 95% CI, 1.32-2.16; *P* < .001), composite MALE or POD (OR, 1.50; 95% CI, 0.96-2.33; *P* = .075), major reintervention (OR, 1.95; 95% CI, 1.42-2.67; *P* < .001), and MACE (OR, 4.14; 95% CI, 2.51-6.82; *P* < .001). The OPEN-Other group was associated with similar odds of POD (OR, 1.24; 95% CI, 0.91-1.70; *P* = .17). Results may be seen in Supplemental Table S11.

### Sensitivity analysis: IPWRA—Femoral-tibial

Compared to the OPEN-GSV group, the ENDO group was associated with similar risks of major amputation (RR, 1.17; 95% CI, 0.85-1.60; *P* = .33), MALE (RR, 1.06; 95% CI, 0.84-1.33; *P* = .63), MALE or POD (RR, 1.02; 95% CI, 0.83-1.25; *P* = .86), POD (RR, 0.74; 95% CI, 0.48-1.13; *P* = .16), and major reintervention (RR, 0.87; 95% CI, 0.63-1.19; *P* = .38) but a lower risk of MACE (RR, 0.33; 95% CI, 0.20-0.54; *P* < .001).

Compared to the OPEN-Other group, the ENDO group was associated with similar risks of major amputation (RR, 0.79; 95% CI, 0.57-1.11; *P* = .18), MALE (RR, 0.81; 95% CI, 0.63-1.03; *P* = .09), MALE or POD (RR, 0.82; 95% CI, 0.66-1.02; *P* = .07), and POD (RR, 0.78; 95% CI, 0.47-1.27; *P* = .31) but a lower risk of major reintervention (RR, 0.68; 95% CI, 0.48-0.95; *P* = .025) and MACE (RR, 0.27; 95% CI, 0.16-0.45; *P* < .001).

Results from these analyses are displayed in Supplemental Table S12.

### Patient characteristics: Popliteal-tibial

We identified a total of 2769 patients in the ENDO group, 1037 in the OPEN-GSV group, and 269 patients in the OPEN-Other group. The average age was 69.4 ± 12.2 years for the ENDO group, 67.8 ± 12.2 years for the OPEN-GSV group (ENDO vs OPEN-GSV: *P* = .001), and 69.0 ± 10.1 years in the OPEN-Other group (vs ENDO: *P* = .60). There was a higher proportion of male patients compared to female patients in all 3 groups (ENDO vs OPEN-GSV: 68% vs 72.9%; *P* = .013; ENDO vs OPEN-Other: 68% vs 68.4%; *P* = .88). More data on patient characteristics can be seen in Supplemental Tables S12-S15.

### Multivariable regression: Popliteal-tibial

Compared to the ENDO group, the OPEN-GSV group was associated with similar rates of major amputations (OR, 0.77; 95% CI, 0.51-1.16; *P* = .21), MALE (OR, 0.89; 95% CI, 0.66-1.20; *P* = .444), POD (OR, 1.34; 95% CI, 0.84-2.12; *P* = .215), composite MALE or POD (OR, 1.02; 95% CI, 0.78-1.32; *P* = .907), major reintervention (OR, 1.17; 95% CI, 0.79-1.73; *P* = .446). OPEN-GSV was associated with higher odds of MACE compared to ENDO (OR, 3.71; 95% CI, 2.14-6.42; *P* < .001).

Compared to the ENDO group, the OPEN-Other group was associated with similar rates of major amputation (OR, 0.63; 95% CI, 0.28-1.40; *P* = .255), MALE (OR, 0.87; 95% CI, 0.51-1.46; *P* = .588), composite MALE or POD (OR, 1.12; 95% CI, 0.72-1.74; *P* = .606), major reintervention (1.05; 95% CI, 0.56-2.00; *P* = .872). The OPEN-Other group was associated with higher odds of POD (OR, 2.45; 95% CI, 1.22-4.89; *P* = .011) and MACE (OR, 2.62; 95% CI, 1.02-6.72; *P* = .045). Results may be seen in Supplemental Table S16.

### Sensitivity analysis: IPWRA—Popliteal-tibial

Compared to the OPEN-GSV group, the ENDO group was associated with similar risks of major amputation (RR, 1.21; 95% CI, 0.84-1.76; *P* = 0.31), MALE (RR, 1.16; 95% CI, 0.88-1.54; *P* = .30), MALE or POD (RR, 1.04; 95% CI, 0.82-1.31; *P* = .75), POD (RR, 0.82; 95% CI, 0.52-1.29; *P* = .38), and major reintervention (RR, 0.89; 95% CI, 0.61-1.31; *P* = .55) but a lower risk of MACE (RR, 0.31; 95% CI, 0.20-0.49; *P* < .001).

Compared to the OPEN-Other group, the ENDO group was associated with similar risks of major amputation (RR, 1.27; 95% CI, 0.62-2.62; *P* = .51), MALE (RR, 1.00; 95% CI, 0.60-1.66; *P* = 1.00), MALE or POD (RR, 0.79; 95% CI, 0.54-1.15; *P* = .22), and major reintervention (RR, 0.63; 95% CI, 0.33-1.21; *P* = .17) but a lower risk of mortality (RR, 0.56; 95% CI, 0.33-0.96; *P* = .036) and a trend toward a lower risk of MACE (RR, 0.44; 95% CI, 0.18-1.03; *P* = .058).

Results from these analyses are displayed in Supplemental Table S17.

### Secondary outcomes

There was a lower rate of discharge other than home in the ENDO group (20.20%, n = 1777) compared to the OPEN-GSV (35.20%, n = 2356; *P* < .001) and OPEN-Other (38.26%, n = 1576; *P* < .001) groups. There was also a lower rate of bleeding requiring transfusion or secondary procedure in the ENDO group (7.98%, n = 709) compared to the OPEN-GSV (18.30%, n = 1241; *P* < .001) and OPEN-Other (21.73%, n = 913; *P* < .001) groups. The ENDO group had lower rates of wound infections (2.54%, n = 224) compared to the OPEN-GSV (14.57%, n = 988; *P* < .001) and OPEN-Other (13.23%, n = 556; *P* < .001) groups. The ENDO group also had lower rates of MACE (2.18%, n = 194) compared to the OPEN-GSV (3.92%, n = 266) and OPEN-Other (3.95%, n = 165; *P* < .001) groups. There was no difference in 30-day unplanned readmission among the 3 groups (ENDO: 18.62%, n = 1358; OPEN-GSV: 19.28%, n = 1134, *P* = .33; OPEN-Other: 20.17%, n = 740, *P* = .051). The ENDO group had lower rates of serious complications (19.02%, n = 1690) compared to the OPEN-GSV (26.47%, n = 1795; *P* ≤ .001) and OPEN-Other (27.18%, n = 1142; *P* < .001) groups. Additional results can be seen in Table 3.

## Discussion

This study utilized a national surgical database containing data on CLTI interventions from 2014 to 2019 to examine early postoperative outcomes. The ENDO group had similar rates of composite MALE and/or POD at 30 days compared to both the OPEN-GSV and OPEN-Other groups. However, the ENDO group was associated with higher rates of early major amputation and MALE in comparison to the OPEN-GSV group. Patients treated with ENDO had similar rates of major amputations, MALE, composite MALE or POD, and major reintervention compared to the OPEN-Other group. The ENDO group was associated with lower rates of peri-procedural MACE as compared to both the OPEN-GSV and OPEN-Other groups and lower rates of POD as compared to the OPEN-Other group (Central Illustration). These data highlight the importance of optimal patient selection based on surgical risk, disease severity, and conduit availability as suggested in recent practice guidelines.

**Central Illustration:**
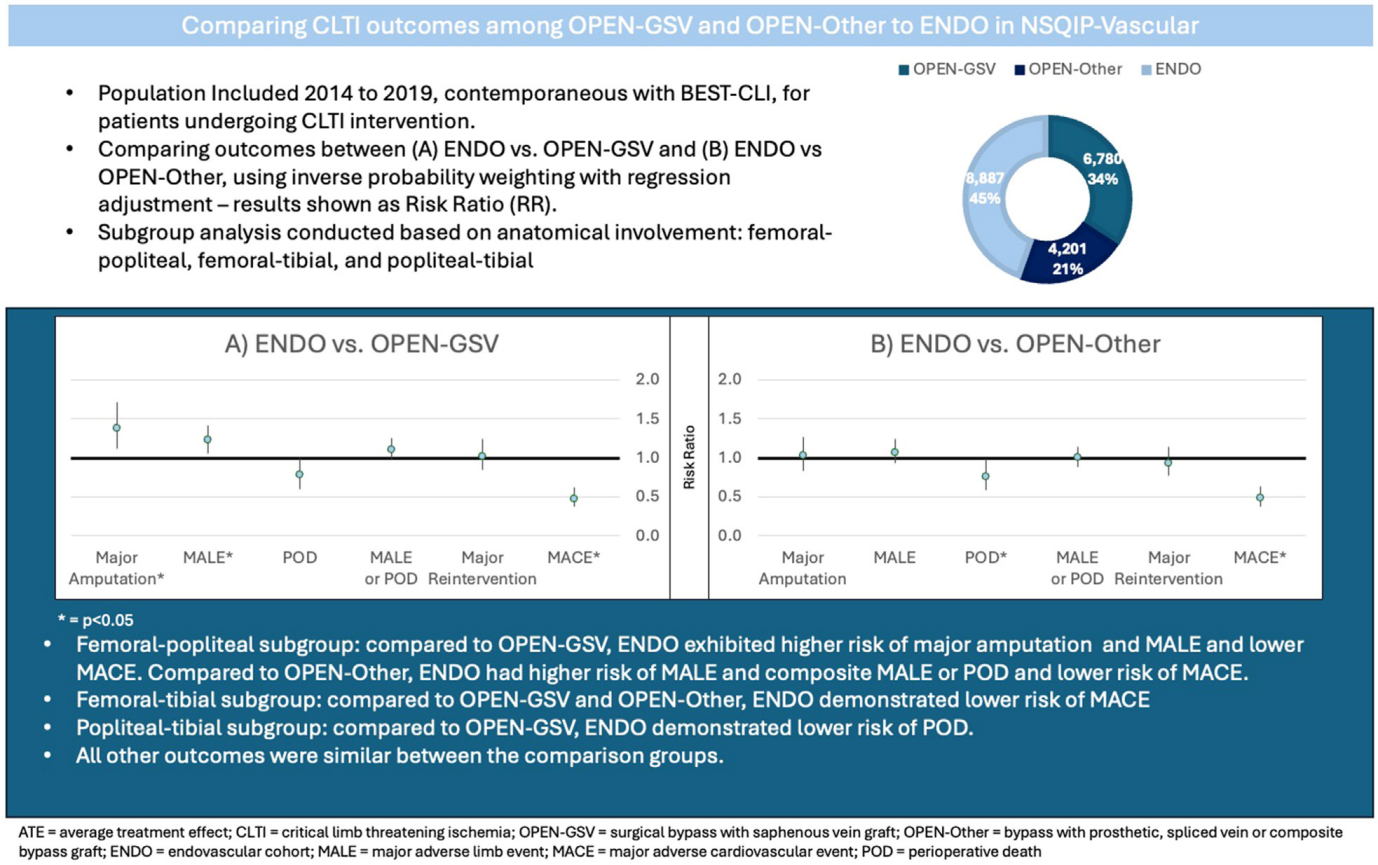
Summary of the study, comparing CLTI outcomes among OPEN-GSV and OPEN-Other to ENDO in National Surgical Quality Improvement Project (NSQIP)-Vascular from 2014 to 2019, in regards to outcomes including major amputation, MALE, POD, composite MALE or POD, major reintervention and MACE.

The BEST-CLI trial was a prospective, multicenter randomized trial comparing open surgical bypass vs endovascular intervention in patients with infrainguinal PAD who were deemed suitable candidates for either treatment. The primary end point of BEST-CLI was the occurrence of MALE or all-cause death at any time; the results significantly favored open bypass among those with an available GSV (cohort 1), whereas there was no significant difference among those requiring an alternative conduit for bypass (cohort 2). In the BEST-CLI, 30-day mortality, MACE, and serious adverse events were not different by intention-to-treat between the open bypass and endovascular arms. Thirty-day major amputation and MALE rates have not been reported at this juncture. In contrast, this contemporary NSQIP analysis demonstrated an increased risk of perioperative MACE in both surgical subgroups but confirms no difference in early mortality between surgical bypass with saphenous vein and endovascular patients. The baseline characteristics between this study and BEST-CLI differ in several important ways including age, gender, race, and comorbidities. Importantly, the BEST trial was specifically designed to include patients who were deemed acceptable candidates for either approach, from both a physiologic and anatomic perspective. The individual variables that differed between these study populations may drive clinical decisions and impact the outcomes. Additionally, the BASIL-2 trial specifically included those that received infrapopliteal intervention with or without an additional more proximal infrainguinal revascularization, which differed from this study, yet demonstrated that endovascular intervention was associated with greater amputation-free survival.^14^ This current study demonstrated that endovascular intervention was associated with a similar treatment effect for amputation-free survival compared to open surgery; this difference may be explained by patient selection. Quandt et al^15^ demonstrated the differences in predictors such as age and average time to treatment between their registry and 4 randomized controlled trials (Virtual International Stroke Trials Archive) regarding outcomes after endovascular treatment for large-vessel occlusion and these differences drove the associations with outcomes.^16^^,^^17^

Latz et al^18^ analyzed 30-day outcomes for endovascular intervention compared to surgical bypass for CLTI in NSQIP, which demonstrated that endovascular intervention was associated with higher odds of MALE and major amputation and lower odds of MACE. In comparison to this current study, the effect may be explained by the difference in groups analyzed whereas the current analysis compared endovascular intervention to GSV and other conduit separately.

In comparison to the results reported by intention-to-treat from BEST-CLI, the data reported here show slightly higher overall rates of POD, and similar rates of early MACE. As-treated results from BEST-CLI, including early periprocedural outcomes, have not been reported yet. However, it will be difficult to directly compare results from this randomized trial to the NSQIP cohorts, because patients enrolled in BEST were selected by the investigators to have equipoise for both treatment options. Indeed, there are numerous differences in the baseline patient characteristics between the BEST-CLI and NSQIP cohorts. Nonetheless, the present data provide an important contemporary context of unselected patients undergoing revascularization for CLTI. Similar to BEST-CLI, these data demonstrate the superiority of OPEN-GSV and the equivalence between ENDO and OPEN-Other for limb-related outcomes (major amputation and MALE).

Robinson et al^19^ highlighted that contemporary real-world practice captured in NSQIP failed to meet the ideal Society of Vascular Surgery’s 2009 Objective Performance Goals.^20^ This example makes clear the importance of population differences between trials and real-world data. Additionally, the rates of adverse events and complications in the Robinson et al’s^19^ study are larger than the rates found in our cohort, which may reveal a significant change in clinical practice, development in intervention, and/or patient selection over the years.^1^^,^^21^^,^^22^ Based on these differences between real-world data and RCT, there is a need for standardization of reporting variables and analyses. Although this current study sheds some light on the differences between real-world data and RCT results, we acknowledge the importance of randomized clinical trial data to remove as much clinician bias as possible to better understand differences in treatment. Conte et al^5^ provided guidelines based on the best available data regarding the method of revascularization for CLTI, stating that bypass may be preferred for average-risk patients with high-complexity disease whereas endovascular intervention would be useful for high-risk patients with less complex anatomy.^5^ We await additional analyses to better understand the results highlighted in BEST-CLI.^9^ Several important questions include the anatomic patterns of disease enrolled, results from as-treated analyses, insights on technical failures, the impact of medical therapy after revascularization, subjects lost to follow-up, and imaging surveillance after the procedure.

### Strengths and limitations

This current study utilized a national surgical database that captured vascular-specific risk factors and outcomes within the same years that the BEST-CLI trial was conducted. Additionally, the objective of NSQIP is prospective data collection to better understand quality improvement factors and 30-day outcomes compared to other national databases based on administrative billing codes and behaviors. This study comes with limitations. Outcomes beyond 30 days are not captured in this database and therefore, long-term follow-up information cannot be compared to the clinical trial. Observational studies such as ours include treatment equipoise, unmeasured confounders that are not able to be accounted for, and possibly measured confounders that may not be fully adjusted. There is also a lack of recording of Rutherford Classification, lesion length, anatomical markers, WIfI stage, and Wegner Classification, which are all captured in the BEST-CLI trial.^9^ Additionally, results in the BEST-CLI study were reported using an “intention-to-treat” analysis whereas NSQIP’s results were analyzed “as-treated.”

## Conclusion

The composite end point of 30-day MALE and POD was similar between endovascular treatment and surgical bypass in this study. Differences in early adverse limb (major amputation) vs systemic (MACE) events highlight the importance of patient selection and the complementary roles of endovascular and surgical revascularization in patients with CLTI.
