# Supplementary material for: Perioperative Events Following Open Versus Endovascular Revascularization for Chronic Limb-Threatening Ischemia: An NSQIP Analysis
Source: J Soc Cardiovasc Angiogr Interv. 2025 Apr 1;4(5):102579. doi: 10.1016/j.jscai.2025.102579 (PMC12126078; doi:10.1016/j.jscai.2025.102579)
Supplement: Supplemental Tables [file mmc1.docx]

**Supplemental Table S1:** Regression Analysis of 30-day outcomes

| **Major Amputation (Transtibial or Proximal)** | | | | | |
| --- | --- | --- | --- | --- | --- |
|  | Odds Ratio | 95% lower bound | 95% upper bound | | p-value |
| **Procedure Type** |  |  |  | |  |
| ENDO | 1.000 |  |  | |  |
| OPEN-GSV | 0.737 | 0.605 | 0.897 | | 0.002 |
| OPEN-Other | 0.993 | 0.812 | 1.215 | | 0.948 |
| **Age** | 0.990 | 0.982 | 0.998 | | 0.014 |
| **Gender** |  |  |  | |  |
| female | 1.000 |  |  | |  |
| male | 1.040 | 0.882 | 1.226 | | 0.644 |
| **BMI** | 1.003 | 0.990 | 1.016 | | 0.676 |
| **Race** |  |  |  | |  |
| White | 1.000 |  |  | |  |
| Black or African American | 1.350 | 1.137 | 1.603 | | <0.001 |
| American Indian or Alaska Native | 1.531 | 0.604 | 3.884 | | 0.369 |
| Asian | 0.850 | 0.425 | 1.698 | | 0.645 |
| Native Hawaiian or Pacific Islander | 0.752 | 0.097 | 5.821 | | 0.785 |
| **High Risk Factors, Physiologic** | 1.056 | 0.853 | 1.307 | | 0.620 |
| **High Risk Factors, Anatomic** |  |  |  | |  |
| None | 1.000 |  |  | |  |
| Prior Bypass | 1.787 | 1.470 | 2.171 | | <0.001 |
| Prior Endovascular Intervention | 0.982 | 0.788 | 1.224 | | 0.872 |
| **Pre-procedural Antiplatelet Medication** | 0.750 | 0.616 | 0.913 | | 0.004 |
| **Pre-procedural Medication-Statin** | 0.773 | 0.648 | 0.922 | | 0.004 |
| **Anatomic Location** |  |  |  | |  |
| Femoral and/or Popliteal | 1.000 |  |  | |  |
| Tibial/Distal | 1.298 | 1.105 | 1.526 | | 0.002 |
| **Functional Health Status** |  |  |  | |  |
| Independent | 1.000 |  |  | |  |
| Partially Dependent | 1.220 | 0.972 | 1.532 | | 0.087 |
| Totally Dependent | 1.581 | 0.911 | 2.744 | | 0.104 |
| **Diabetes** |  |  |  | |  |
| None | 1.000 |  |  | |  |
| Non-Insulin Dependent | 0.892 | 0.707 | 1.125 | | 0.334 |
| Insulin Dependent | 1.008 | 0.837 | 1.215 | | 0.930 |
| **Current Smoker within 1 year** | 0.777 | 0.643 | 0.939 | | 0.009 |
| **Ascites** | 3.839 | 1.490 | 9.894 | | 0.005 |
| **CHF with prior 30 days** | 1.092 | 0.775 | 1.538 | | 0.614 |
| **Acute Renal Failure (Pre-Op)** | 1.372 | 0.877 | 2.147 | | 0.166 |
| **Currently on Dialysis** | 1.658 | 1.291 | 2.128 | | <0.001 |
| **Disseminated Cancer** | 2.189 | 1.027 | 4.666 | | 0.043 |
| **Open wound/wound infection** | 0.915 | 0.776 | 1.078 | | 0.288 |
| **Steroid use for chronic condition** | 1.365 | 1.030 | 1.809 | | 0.030 |
| **>10% loss body weight in last 6 months** | 2.298 | 1.467 | 3.599 | | <0.001 |
| **Preop Transfusion with 72 hrs prior to surgery** | 1.191 | 0.768 | 1.846 | | 0.435 |
| **Systemic Sepsis** |  |  |  | |  |
| None | 1.000 |  |  | |  |
| SIRS | 2.684 | 2.093 | 3.442 | | <0.001 |
| Sepsis | 4.602 | 3.202 | 6.614 | | <0.001 |
| Septic Shock | 2.057 | 0.259 | 16.353 | | 0.495 |
| **Major Adverse Limb Event** | | | | | |
|  | Odds Ratio | 95% lower bound | 95% upper bound | p-value | |
| **Procedure Type** |  |  |  |  | |
| ENDO | 1.000 |  |  |  | |
| OPEN-GSV | 0.837 | 0.727 | 0.963 | 0.013 | |
| OPEN-Other | 0.973 | 0.838 | 1.129 | 0.715 | |
| **Age** | 0.992 | 0.986 | 0.998 | 0.007 | |
| **Gender** |  |  |  |  | |
| female | 1.000 |  |  |  | |
| male | 0.978 | 0.867 | 1.102 | 0.710 | |
| **BMI** | 1.007 | 0.998 | 1.017 | 0.140 | |
| **Race** |  |  |  |  | |
| White | 1.000 |  |  |  | |
| Black or African American | 1.141 | 1.001 | 1.300 | 0.048 | |
| American Indian or Alaska Native | 1.076 | 0.487 | 2.377 | 0.856 | |
| Asian | 1.017 | 0.635 | 1.630 | 0.944 | |
| Native Hawaiian or Pacific Islander | 0.429 | 0.057 | 3.249 | 0.413 | |
| **High Risk Factors, Physiologic** | 1.108 | 0.950 | 1.292 | 0.193 | |
| **High Risk Factors, Anatomic** |  |  |  |  | |
| None | 1.000 |  |  |  | |
| Prior Bypass | 1.845 | 1.600 | 2.126 | <0.001 | |
| Prior Endovascular Intervention | 1.191 | 1.022 | 1.387 | 0.025 | |
| **Pre-procedural Antiplatelet Medication** | 0.765 | 0.660 | 0.888 | <0.001 | |
| **Pre-procedural Medication-Statin** | 0.950 | 0.831 | 1.086 | 0.452 | |
| **Anatomic Location** |  |  |  |  | |
| Femoral and/or Popliteal | 1.000 |  |  |  | |
| Tibial/Distal | 1.276 | 1.133 | 1.437 | <0.001 | |
| **Functional Health Status** |  |  |  |  | |
| Independent | 1.000 |  |  |  | |
| Partially Dependent | 1.252 | 1.055 | 1.486 | 0.010 | |
| Totally Dependent | 1.187 | 0.738 | 1.909 | 0.480 | |
| **Diabetes** |  |  |  |  | |
| None | 1.000 |  |  |  | |
| Non-Insulin Dependent | 0.890 | 0.754 | 1.051 | 0.170 | |
| Insulin Dependent | 0.976 | 0.850 | 1.121 | 0.728 | |
| **Current Smoker within 1 year** | 0.964 | 0.842 | 1.104 | 0.600 | |
| **Ascites** | 3.232 | 1.346 | 7.763 | 0.009 | |
| **Acute Renal Failure (Pre-Op)** | 1.328 | 0.908 | 1.943 | 0.144 | |
| **Currently on Dialysis** | 1.237 | 1.014 | 1.509 | 0.036 | |
| **Disseminated Cancer** | 1.377 | 0.702 | 2.701 | 0.352 | |
| **Open wound/wound infection** | 0.875 | 0.777 | 0.986 | 0.029 | |
| **Steroid use for chronic condition** | 1.346 | 1.087 | 1.666 | 0.006 | |
| **>10% loss body weight in last 6 months** | 1.832 | 1.250 | 2.684 | 0.002 | |
| **Systemic Sepsis** |  |  |  |  | |
| None | 1.000 |  |  |  | |
| SIRS | 2.029 | 1.648 | 2.499 | <0.001 | |
| Sepsis | 2.845 | 2.045 | 3.958 | <0.001 | |
| Septic Shock | 2.265 | 0.491 | 10.454 | 0.295 | |
| **Hypertension requiring medications** | 0.942 | 0.805 | 1.102 | 0.456 | |
| **Perioperative Death** | | | | | |
|  | Odds Ratio | 95% lower bound | 95% upper bound | p-value | |
| **Procedure Type** |  |  |  |  | |
| ENDO | 1.000 |  |  |  | |
| OPEN-GSV | 1.213 | 0.960 | 1.533 | 0.105 | |
| OPEN-Other | 1.368 | 1.072 | 1.745 | 0.012 | |
| **Age** | 1.048 | 1.037 | 1.060 | <0.001 | |
| **Gender** |  |  |  |  | |
| female | 1.000 |  |  |  | |
| male | 0.976 | 0.802 | 1.187 | 0.805 | |
| **BMI** | 0.996 | 0.980 | 1.013 | 0.684 | |
| **High Risk Factors, Physiologic** | 1.534 | 1.200 | 1.962 | 0.001 | |
| **High Risk Factors, Anatomic** |  |  |  |  | |
| None | 1.000 |  |  |  | |
| Prior Bypass | 0.822 | 0.616 | 1.098 | 0.185 | |
| Prior Endovascular Intervention | 1.058 | 0.831 | 1.347 | 0.646 | |
| **Pre-procedural Antiplatelet Medication** | 1.239 | 0.951 | 1.614 | 0.113 | |
| **Pre-procedural Medication-Statin** | 0.813 | 0.654 | 1.009 | 0.060 | |
| **Anatomic Location** |  |  |  |  | |
| Femoral and/or Popliteal | 1.000 |  |  |  | |
| Tibial/Distal | 0.944 | 0.774 | 1.152 | 0.573 | |
| **Functional Health Status** |  |  |  |  | |
| Independent | 1.000 |  |  |  | |
| Partially Dependent | 1.181 | 0.920 | 1.514 | 0.191 | |
| Totally Dependent | 2.203 | 1.335 | 3.638 | 0.002 | |
| **Diabetes** |  |  |  |  | |
| None | 1.000 |  |  |  | |
| Non-Insulin Dependent | 0.994 | 0.758 | 1.303 | 0.967 | |
| Insulin Dependent | 1.080 | 0.857 | 1.362 | 0.514 | |
| **Current Smoker within 1 year** | 0.847 | 0.651 | 1.102 | 0.216 | |
| **Ascites** | 6.179 | 2.434 | 15.688 | <0.001 | |
| **CHF with prior 30 days** | 2.245 | 1.670 | 3.019 | <0.001 | |
| **Acute Renal Failure (Pre-Op)** | 0.853 | 0.510 | 1.427 | 0.545 | |
| **Currently on Dialysis** | 3.168 | 2.452 | 4.093 | <0.001 | |
| **Disseminated Cancer** | 5.926 | 3.142 | 11.178 | <0.001 | |
| **Open wound/wound infection** | 0.983 | 0.806 | 1.198 | 0.863 | |
| **Steroid use for chronic condition** | 1.371 | 0.982 | 1.912 | 0.064 | |
| **>10% loss body weight in last 6 months** | 2.214 | 1.335 | 3.669 | 0.002 | |
| **Preop Transfusion with 72 hrs prior to surgery** | 1.327 | 0.817 | 2.154 | 0.253 | |
| **Systemic Sepsis** |  |  |  |  | |
| None | 1.000 |  |  |  | |
| SIRS | 2.573 | 1.911 | 3.465 | <0.001 | |
| Sepsis | 3.755 | 2.334 | 6.042 | <0.001 | |
| Septic Shock | 16.289 | 4.803 | 55.245 | <0.001 | |
| **Hypertension requiring medications** | 0.901 | 0.678 | 1.198 | 0.474 | |
| **Dyspnea** |  |  |  |  | |
| No | 1.000 |  |  |  | |
| Moderate Exertion | 1.401 | 1.072 | 1.829 | 0.013 | |
| At Rest | 2.136 | 1.131 | 4.034 | 0.019 | |
| **Pre-procedural Medication-Beta Blocker** | 1.155 | 0.927 | 1.438 | 0.199 | |
| **History of Severe COPD** | 1.323 | 1.009 | 1.735 | 0.043 | |
| **MALE or Perioperative Death** | | | | | |
|  | Odds Ratio | 95% lower bound | 95% upper bound | p-value | |
| **Procedure Type** |  |  |  |  | |
| ENDO | 1.000 |  |  |  | |
| OPEN-GSV | 0.909 | 0.800 | 1.032 | 0.140 | |
| OPEN-Other | 1.029 | 0.899 | 1.178 | 0.679 | |
| **Age** | 1.001 | 0.995 | 1.006 | 0.811 | |
| **Gender** |  |  |  |  | |
| female | 1.000 |  |  |  | |
| male | 0.961 | 0.863 | 1.072 | 0.477 | |
| **BMI** | 1.004 | 0.996 | 1.013 | 0.318 | |
| **Race** |  |  |  |  | |
| White | 1.000 |  |  |  | |
| Black or African American | 1.032 | 0.914 | 1.165 | 0.612 | |
| American Indian or Alaska Native | 0.910 | 0.429 | 1.929 | 0.806 | |
| Asian | 0.841 | 0.540 | 1.310 | 0.444 | |
| Native Hawaiian or Pacific Islander | 0.614 | 0.141 | 2.676 | 0.516 | |
| **High Risk Factors, Physiologic** | 1.223 | 1.065 | 1.404 | 0.004 | |
| **High Risk Factors, Anatomic** |  |  |  |  | |
| None | 1.000 |  |  |  | |
| Prior Bypass | 1.618 | 1.418 | 1.848 | <0.001 | |
| Prior Endovascular Intervention | 1.166 | 1.017 | 1.337 | 0.028 | |
| **Pre-procedural Antiplatelet Medication** | 0.826 | 0.721 | 0.947 | 0.006 | |
| **Pre-procedural Medication-Statin** | 0.921 | 0.816 | 1.039 | 0.180 | |
| **Anatomic Location** |  |  |  |  | |
| Femoral and/or Popliteal | 1.000 |  |  |  | |
| Tibial/Distal | 1.187 | 1.066 | 1.322 | 0.002 | |
| **Functional Health Status** |  |  |  |  | |
| Independent | 1.000 |  |  |  | |
| Partially Dependent | 1.225 | 1.052 | 1.426 | 0.009 | |
| Totally Dependent | 1.771 | 1.229 | 2.552 | 0.002 | |
| **Diabetes** |  |  |  |  | |
| None | 1.000 |  |  |  | |
| Non-Insulin Dependent | 0.892 | 0.768 | 1.036 | 0.136 | |
| Insulin Dependent | 0.969 | 0.856 | 1.097 | 0.624 | |
| **Current Smoker within 1 year** | 0.921 | 0.812 | 1.044 | 0.199 | |
| **Ascites** | 5.518 | 2.453 | 12.411 | <0.001 | |
| **CHF with prior 30 days** | 1.397 | 1.117 | 1.747 | 0.003 | |
| **Acute Renal Failure (Pre-Op)** | 1.225 | 0.874 | 1.715 | 0.239 | |
| **Currently on Dialysis** | 1.528 | 1.288 | 1.814 | <0.001 | |
| **Disseminated Cancer** | 2.501 | 1.491 | 4.196 | <0.001 | |
| **Steroid use for chronic condition** | 1.342 | 1.107 | 1.627 | 0.003 | |
| **>10% loss body weight in last 6 months** | 1.951 | 1.388 | 2.742 | <0.001 | |
| **Preop Transfusion with 72 hrs prior to surgery** | 1.156 | 0.841 | 1.588 | 0.372 | |
| **Systemic Sepsis** |  |  |  |  | |
| None | 1.000 |  |  |  | |
| SIRS | 2.212 | 1.835 | 2.666 | <0.001 | |
| Sepsis | 2.952 | 2.181 | 3.995 | <0.001 | |
| Septic Shock | 6.990 | 2.191 | 22.307 | 0.001 | |
| **Hypertension requiring medications** | 0.958 | 0.829 | 1.107 | 0.563 | |
| **Dyspnea** |  |  |  |  | |
| No | 1.000 |  |  |  | |
| Moderate Exertion | 0.998 | 0.843 | 1.180 | 0.977 | |
| At Rest | 1.335 | 0.813 | 2.193 | 0.253 | |
| **Major Reintervention of Treated Segment** | | | | | |
|  | Odds Ratio | 95% lower bound | 95% upper bound | p-value | |
| **Procedure Type** |  |  |  |  | |
| ENDO | 1.000 |  |  |  | |
| OPEN-GSV | 1.008 | 0.844 | 1.203 | 0.931 | |
| OPEN-Other | 1.102 | 0.912 | 1.331 | 0.316 | |
| **Age** | 0.995 | 0.987 | 1.002 | 0.163 | |
| **Gender** |  |  |  |  | |
| female | 1.000 |  |  |  | |
| male | 0.910 | 0.782 | 1.059 | 0.222 | |
| **BMI** | 1.009 | 0.997 | 1.021 | 0.133 | |
| **Race** |  |  |  |  | |
| White | 1.000 |  |  |  | |
| Black or African American | 0.959 | 0.807 | 1.141 | 0.637 | |
| American Indian or Alaska Native | 1.113 | 0.403 | 3.074 | 0.837 | |
| Asian | 1.208 | 0.682 | 2.137 | 0.517 | |
| Native Hawaiian or Pacific Islander | 1.000 |  |  |  | |
| **High Risk Factors, Physiologic** | 1.093 | 0.897 | 1.333 | 0.378 | |
| **High Risk Factors, Anatomic** |  |  |  |  | |
| None | 1.000 |  |  |  | |
| Prior Bypass | 1.894 | 1.586 | 2.262 | <0.001 | |
| Prior Endovascular Intervention | 1.356 | 1.121 | 1.641 | 0.002 | |
| **Pre-procedural Antiplatelet Medication** | 0.728 | 0.602 | 0.880 | 0.001 | |
| **Pre-procedural Medication-Statin** | 1.098 | 0.923 | 1.307 | 0.292 | |
| **Anatomic Location** |  |  |  |  | |
| Femoral and/or Popliteal | 1.000 |  |  |  | |
| Tibial/Distal | 1.265 | 1.087 | 1.472 | 0.002 | |
| **Diabetes** |  |  |  |  | |
| None | 1.000 |  |  |  | |
| Non-Insulin Dependent | 0.904 | 0.736 | 1.111 | 0.338 | |
| Insulin Dependent | 0.901 | 0.754 | 1.077 | 0.254 | |
| **Current Smoker within 1 year** | 1.084 | 0.915 | 1.284 | 0.351 | |
| **Acute Renal Failure (Pre-Op)** | 1.113 | 0.619 | 2.002 | 0.722 | |
| **Currently on Dialysis** | 0.917 | 0.690 | 1.218 | 0.549 | |
| **Open wound/wound infection** | 0.841 | 0.723 | 0.979 | 0.026 | |
| **Systemic Sepsis** |  |  |  |  | |
| None | 1.000 |  |  |  | |
| SIRS | 1.365 | 1.004 | 1.855 | 0.047 | |
| Sepsis | 1.357 | 0.781 | 2.356 | 0.279 | |
| Septic Shock | 1.797 | 0.230 | 14.058 | 0.577 | |
| **Hypertension requiring medications** | 0.956 | 0.783 | 1.168 | 0.661 | |
| **Major Adverse Cardiovascular Event** | | | | | |
|  | Odds Ratio | 95% lower bound | 95% upper bound | p-value | |
| **Procedure Type** |  |  |  |  | |
| ENDO | 1.000 |  |  |  | |
| OPEN-GSV | 2.071 | 1.597 | 2.686 | <0.001 | |
| OPEN-Other | 2.150 | 1.642 | 2.814 | <0.001 | |
| **Age** | 1.011 | 1.000 | 1.023 | 0.058 | |
| **Gender** |  |  |  |  | |
| female | 1.000 |  |  |  | |
| male | 0.961 | 0.773 | 1.195 | 0.722 | |
| **BMI** | 0.995 | 0.977 | 1.013 | 0.574 | |
| **Race** |  |  |  |  | |
| White | 1.000 |  |  |  | |
| Black or African American | 0.778 | 0.599 | 1.009 | 0.058 | |
| American Indian or Alaska Native | 0.970 | 0.234 | 4.025 | 0.967 | |
| Asian | 1.082 | 0.498 | 2.349 | 0.842 | |
| Native Hawaiian or Pacific Islander | 1.000 |  |  |  | |
| **High Risk Factors, Physiologic** | 1.155 | 0.880 | 1.517 | 0.299 | |
| **High Risk Factors, Anatomic** |  |  |  |  | |
| None | 1.000 |  |  |  | |
| Prior Bypass | 0.953 | 0.718 | 1.265 | 0.738 | |
| Prior Endovascular Intervention | 1.043 | 0.799 | 1.360 | 0.757 | |
| **Pre-procedural Antiplatelet Medication** | 1.266 | 0.917 | 1.748 | 0.151 | |
| **Pre-procedural Medication-Statin** | 1.127 | 0.867 | 1.463 | 0.372 | |
| **Anatomic Location** |  |  |  |  | |
| Femoral and/or Popliteal | 1.000 |  |  |  | |
| Tibial/Distal | 0.969 | 0.780 | 1.203 | 0.773 | |
| **Diabetes** |  |  |  |  | |
| None | 1.000 |  |  |  | |
| Non-Insulin Dependent | 1.231 | 0.913 | 1.660 | 0.173 | |
| Insulin Dependent | 1.497 | 1.166 | 1.924 | 0.002 | |
| **Current Smoker within 1 year** | 0.781 | 0.601 | 1.014 | 0.063 | |
| **CHF with prior 30 days** | 1.022 | 0.649 | 1.610 | 0.924 | |
| **Currently on Dialysis** | 1.742 | 1.257 | 2.415 | 0.001 | |
| **Disseminated Cancer** | 1.932 | 0.686 | 5.446 | 0.213 | |
| **>10% loss body weight in last 6 months** | 2.064 | 1.084 | 3.929 | 0.027 | |
| **Systemic Sepsis** |  |  |  |  | |
| None | 1.000 |  |  |  | |
| SIRS | 1.614 | 1.092 | 2.386 | 0.016 | |
| Sepsis | 0.727 | 0.267 | 1.984 | 0.534 | |
| Septic Shock | 6.566 | 1.356 | 31.793 | 0.019 | |
| **Hypertension requiring medications** | 1.617 | 1.101 | 2.377 | 0.014 | |
| **Dyspnea** |  |  |  |  | |
| No | 1.000 |  |  |  | |
| Moderate Exertion | 1.501 | 1.117 | 2.017 | 0.007 | |
| At Rest | 1.314 | 0.469 | 3.686 | 0.603 | |
| **Pre-procedural Medication-Beta Blocker** | 1.275 | 0.997 | 1.632 | 0.053 | |
| **Bleeding Disorder** | 1.226 | 0.981 | 1.533 | 0.073 | |

**Supplemental Table S2:** Inverse-propensity weighted with regression adjustment analysis, by symptomatology, rest pain and tissue loss.

| **Rest Pain** | | | | |
| --- | --- | --- | --- | --- |
| **ENDO vs. OPEN-GSV** | | | | |
| **Variable** | **Risk Ratio** | **95% lower bound** | **95% upper bound** | **p-value** |
| Major Amputation | 1.67 | 1.14 | 2.46 | 0.009 |
| MALE | 1.45 | 1.15 | 1.84 | 0.002 |
| POD | 0.88 | 0.55 | 1.42 | 0.60 |
| MALE or POD | 1.32 | 1.06 | 1.64 | 0.012 |
| Major Reintervention | 1.33 | 1.00 | 1.77 | 0.051 |
| MACE | 0.75 | 0.47 | 1.19 | 0.21 |
| **ENDO vs. OPEN-Other** | | | | |
| **Variable** | **Risk Ratio** | **95% lower bound** | **95% upper bound** | **p-value** |
| Major Amputation | 1.24 | 0.87 | 1.75 | 0.23 |
| MALE | 1.25 | 0.99 | 1.57 | 0.055 |
| POD | 0.92 | 0.58 | 1.44 | 0.71 |
| MALE or POD | 1.16 | 0.95 | 1.42 | 0.15 |
| Major Reintervention | 1.31 | 0.98 | 1.74 | 0.07 |
| MACE | 0.66 | 0.43 | 1.01 | 0.055 |
| **Tissue Loss** | | | | |
| **ENDO vs. OPEN-GSV** | | | | |
| **Variable** | **Risk Ratio** | **95% lower bound** | **95% upper bound** | **p-value** |
| Major Amputation | 1.27 | 0.99 | 1.63 | 0.06 |
| MALE | 1.15 | 0.95 | 1.38 | 0.14 |
| POD | 0.71 | 0.53 | 0.97 | 0.028 |
| MALE or POD | 1.02 | 0.88 | 1.20 | 0.77 |
| Major Reintervention | 0.88 | 0.68 | 1.13 | 0.30 |
| MACE | 0.39 | 0.29 | 0.53 | <0.001 |
| **ENDO vs. OPEN-Other** | | | | |
| **Variable** | **Risk Ratio** | **95% lower bound** | **95% upper bound** | **p-value** |
| Major Amputation | 1.10 | 0.86 | 1.39 | 0.46 |
| MALE | 1.04 | 0.87 | 1.25 | 0.65 |
| POD | 0.64 | 0.48 | 0.86 | 0.003 |
| MALE or POD | 0.94 | 0.81 | 1.10 | 0.46 |
| Major Reintervention | 0.79 | 0.61 | 1.01 | 0.06 |
| MACE | 0.44 | 0.32 | 0.59 | <0.001 |

**Supplemental Table S3:** Patient Demographics**,** Comparison of OPEN-GSV vs. ENDO vs. OPEN-Other, Femoral-Popliteal

|  | **Procedure Type** | | | **P-value** | | |
| --- | --- | --- | --- | --- | --- | --- |
|  | **OPEN-GSV**  **(n=3,085)** | **ENDO**  **(n=6,118)** | **OPEN-Other**  **(n=2,434)** | **Overall** | **ENDO vs. OPEN-GSV** | **ENDO vs. OPEN-Other** |
| Age | 67.2 (11.1) | 69.7 (11.8) | 69.0 (10.7) | <0.001 | <0.001 | 0.009 |
| BMI | 27.567 (5.967) | 28.086 (6.502) | 26.980 (6.017) | <0.001 | <0.001 | <0.001 |
| Female Sex | 1,145 (37.1%) | 2,774 (45.3%) | 984 (40.4%) | <0.001 | <0.001 | <0.001 |
| **RACE** |  |  |  |  |  |  |
| American Indian or Alaska Native | 7 (0.3%) | 31 (0.6%) | 8 (0.4%) | <0.001 | <0.001 | <0.001 |
| Asian | 22 (1.0%) | 103 (2.0%) | 19 (0.9%) |  |  |  |
| Black or African American | 454 (19.9%) | 1,236 (23.5%) | 546 (26.9%) |  |  |  |
| Native Hawaiian or Pacific Islander | 2 (0.1%) | 11 (0.2%) | 1 (0.0%) |  |  |  |
| White | 1,794 (78.7%) | 3,879 (73.7%) | 1,452 (71.7%) |  |  |  |
| **Functional Health Status** |  |  |  |  |  |  |
| Independent | 2,850 (92.7%) | 5,149 (84.6%) | 2,148 (88.9%) | <0.001 | <0.001 | <0.001 |
| Partially Dependent | 212 (6.9%) | 830 (13.6%) | 242 (10.0%) |  |  |  |
| Totally Dependent | 13 (0.4%) | 105 (1.7%) | 26 (1.1%) |  |  |  |
| **DIABETES** |  |  |  |  |  |  |
| None | 1,640 (53.2%) | 2,534 (41.4%) | 1,213 (49.8%) | <0.001 | <0.001 | <0.001 |
| Non-Insulin Dependent | 604 (19.6%) | 1,191 (19.5%) | 456 (18.7%) |  |  |  |
| Insulin Dependent | 841 (27.3%) | 2,393 (39.1%) | 765 (31.4%) |  |  |  |
| **Current Smoker within 1 year** | 1,534 (49.7%) | 1,935 (31.6%) | 1,026 (42.2%) | <0.001 | <0.001 | <0.001 |
| **DYSPNEA** |  |  |  |  |  |  |
| No | 2,781 (90.1%) | 5,398 (88.2%) | 2,149 (88.3%) | 0.036 | 0.012 | 0.857 |
| Moderate Exertion | 286 (9.3%) | 662 (10.8%) | 259 (10.6%) |  |  |  |
| At Rest | 18 (0.6%) | 58 (0.9%) | 26 (1.1%) |  |  |  |
| **Ventilator Dependent** | 1 (0.0%) | 9 (0.1%) | 1 (0.0%) | 0.151 | 0.115 | 0.195 |
| **History of Severe COPD** | 409 (13.3%) | 714 (11.7%) | 385 (15.8%) | <0.001 | 0.028 | <0.001 |
| **Ascites** | 3 (0.1%) | 11 (0.2%) | 4 (0.2%) | 0.63 | 0.337 | 0.877 |
| **CHF with prior 30 days** | 79 (2.6%) | 299 (4.9%) | 120 (4.9%) | <0.001 | <0.001 | 0.934 |
| **Hypertension requiring medications** | 2,449 (79.4%) | 5,191 (84.8%) | 2,054 (84.4%) | <0.001 | <0.001 | 0.594 |
| **Acute Renal Failure (Pre-Op)** | 28 (0.9%) | 94 (1.5%) | 34 (1.4%) | 0.045 | 0.013 | 0.631 |
| **Currently on Dialysis** | 176 (5.7%) | 761 (12.4%) | 221 (9.1%) | <0.001 | <0.001 | <0.001 |
| **Disseminated Cancer** | 12 (0.4%) | 38 (0.6%) | 19 (0.8%) | 0.156 | 0.153 | 0.413 |
| **Open wound/wound infection** | 1,434 (46.5%) | 2,920 (47.7%) | 1,065 (43.8%) | 0.004 | 0.259 | <0.001 |
| **Steroid use for chronic condition** | 155 (5.0%) | 385 (6.3%) | 140 (5.8%) | 0.049 | 0.015 | 0.347 |
| **>10% loss body weight in last 6 months** | 26 (0.8%) | 77 (1.3%) | 46 (1.9%) | 0.003 | 0.073 | 0.027 |
| **Bleeding Disorder** | 585 (22.3%) | 1,697 (34.5%) | 562 (26.7%) | <0.001 | <0.001 | <0.001 |
| **Preop Transfusion with 72 hrs prior to surgery** | 44 (1.4%) | 103 (1.7%) | 65 (2.7%) | 0.001 | 0.353 | 0.003 |
| **Systemic Sepsis** |  |  |  |  |  |  |
| None | 2,941 (95.3%) | 5,714 (93.4%) | 2,311 (94.9%) | 0.001 | <0.001 | 0.06 |
| SIRS | 97 (3.1%) | 311 (5.1%) | 97 (4.0%) |  |  |  |
| Sepsis | 45 (1.5%) | 89 (1.5%) | 25 (1.0%) |  |  |  |
| Septic Shock | 2 (0.1%) | 4 (0.1%) | 1 (0.0%) |  |  |  |

**Supplemental Table S4:** Procedural Data, Comparison of OPEN-GSV vs. ENDO vs. OPEN-Other, Femoral-Popliteal

|  | Procedure Type | | | P-value | | |
| --- | --- | --- | --- | --- | --- | --- |
|  | **OPEN-GSV**  **(n=3,085)** | **ENDO**  **(n=6,118)** | **OPEN-Other**  **(n=2,434)** | **Overall** | **ENDO vs. OPEN-GSV** | **ENDO vs. OPEN-Other** |
| **Symptomatology** |  |  |  |  |  |  |
| Critical limb ischemia: rest pain | 1,363 (44.2%) | 2,215 (36.2%) | 1,169 (48.0%) | <0.001 | <0.001 | <0.001 |
| Critical limb ischemia: tissue loss | 1,722 (55.8%) | 3,903 (63.8%) | 1,265 (52.0%) |  |  |  |
| **High Risk Factors, Physiologic** | 662 (21.6%) | 2,137 (35.1%) | 694 (28.6%) | <0.001 | <0.001 | <0.001 |
| **High Risk Factors, Anatomic** |  |  |  |  |  |  |
| None | 2,000 (64.8%) | 3,836 (62.7%) | 1,355 (55.7%) | <0.001 | 0.033 | <0.001 |
| Prior Bypass | 521 (16.9%) | 1,025 (16.8%) | 622 (25.6%) |  |  |  |
| Prior Endovascular | 564 (18.3%) | 1,257 (20.5%) | 457 (18.8%) |  |  |  |
| **Pre-procedural Antiplatelet Medication** | 2,434 (79.4%) | 5,016 (82.3%) | 2,001 (82.5%) | 0.001 | <0.001 | 0.783 |
| **Pre-procedural Medication-Statin** | 2,174 (70.9%) | 4,374 (71.8%) | 1,836 (75.8%) | <0.001 | 0.366 | <0.001 |
| **Pre-procedural Medication-Beta Blocker** | 1,651 (54.0%) | 3,735 (61.4%) | 1,465 (60.4%) | <0.001 | <0.001 | 0.375 |
| **Overall Procedure** |  |  |  |  |  |  |
| Angioplasty | - | 2,375 (38.8%) | - |  |  |  |
| Atherectomy | - | 1,224 (20.0%) | - |  |  |  |
| Bypass | 3,085 (100.0%) | - | 2,434 (100.0%) |  |  |  |
| Stenting | - | 2,519 (41.2%) | - |  |  |  |
| **ASACLAS** |  |  |  |  |  |  |
| 1-No Disturb | 4 (0.1%) | 11 (0.2%) | 2 (0.1%) | <0.001 | <0.001 | <0.001 |
| 2-Mild Disturb | 82 (2.7%) | 310 (5.4%) | 47 (1.9%) |  |  |  |
| 3-Severe Disturb | 2,146 (69.7%) | 3,782 (65.5%) | 1,599 (65.8%) |  |  |  |
| 4-Life Threat | 842 (27.3%) | 1,664 (28.8%) | 780 (32.1%) |  |  |  |
| 5-Moribund | 5 (0.2%) | 8 (0.1%) | 2 (0.1%) |  |  |  |
| **ANESTHES** |  |  |  |  |  |  |
| None | 0 (0.0%) | 27 (0.4%) | 0 (0.0%) |  |  |  |
| Epidural | 22 (0.7%) | 1 (0.0%) | 14 (0.6%) | <0.001 | <0.001 | <0.001 |
| General | 2,786 (90.3%) | 2,315 (37.8%) | 2,295 (94.3%) |  |  |  |
| Local | 0 (0.0%) | 89 (1.5%) | 0 (0.0%) |  |  |  |
| MAC/IV Sedation | 91 (3.0%) | 3,630 (59.3%) | 45 (1.8%) |  |  |  |
| Other | 8 (0.3%) | 12 (0.2%) | 4 (0.2%) |  |  |  |
| Regional | 10 (0.3%) | 22 (0.4%) | 6 (0.2%) |  |  |  |
| Spinal | 167 (5.4%) | 21 (0.3%) | 69 (2.8%) |  |  |  |
| **Elective Surgery** | 1,760 (57.1%) | 3,141 (51.3%) | 1,345 (55.3%) | <0.001 | <0.001 | <0.001 |
| **OPTIME** | 245.4 (107.3) | 115.5 (73.9) | 208.7 (106.4) | <0.001 | <0.001 | <0.001 |

**Supplemental Table S5:** 30-day outcomes, Comparison of OPEN-GSV vs. ENDO vs. OPEN-Other, Femoral-Popliteal

|  | Procedure Type | | | P-value | | | |  |
| --- | --- | --- | --- | --- | --- | --- | --- | --- |
|  | **OPEN-GSV**  **(n=3,085)** | **ENDO**  **(n=6,118)** | **OPEN-Other**  **(n=2,434)** | | **Overall** | **ENDO vs. OPEN-GSV** | **ENDO vs. OPEN-Other** | |
| **Total Length of Stay, in days** | 8.9 (8.5) | 5.6 (8.1) | 9.0 (8.3) | | <0.001 | <0.001 | <0.001 | |
| **DISCHARGE DESTINATION** |  |  |  | |  |  |  | |
| Against Medical Advice (AMA) | 9 (0.3%) | 12 (0.2%) | 4 (0.2%) | | <0.001 | <0.001 | <0.001 | |
| Expired | 41 (1.3%) | 65 (1.1%) | 45 (1.9%) | |  |  |  | |
| Home | 2,132 (69.6%) | 4,884 (80.2%) | 1,549 (63.8%) | |  |  |  | |
| Hospice | 2 (0.1%) | 20 (0.3%) | 2 (0.1%) | |  |  |  | |
| Multi-level Senior Community | 2 (0.1%) | 0 (0.0%) | 2 (0.1%) | |  |  |  | |
| Rehab | 304 (9.9%) | 362 (5.9%) | 284 (11.7%) | |  |  |  | |
| Separate Acute Care | 71 (2.3%) | 52 (0.9%) | 36 (1.5%) | |  |  |  | |
| Skilled Care, Not Home | 494 (16.1%) | 676 (11.1%) | 497 (20.5%) | |  |  |  | |
| Unskilled Facility Not Home | 8 (0.3%) | 15 (0.2%) | 9 (0.4%) | |  |  |  | |
| **Non-Routine Discharge** | 912 (30.0%) | 1,169 (19.3%) | 840 (35.2%) | | <0.001 | <0.001 | <0.001 | |
| **Untreated Loss of Patency** | 48 (1.6%) | 107 (1.7%) | 34 (1.4%) | | 0.479 | 0.497 | 0.249 | |
| **Bleeding Requiring Transfusion or Secondary Procedure** | 490 (15.9%) | 506 (8.3%) | 479 (19.7%) | | <0.001 | <0.001 | <0.001 | |
| **Myocardial Infarction or Stroke** | 98 (3.2%) | 152 (2.5%) | 80 (3.3%) | | 0.054 | 0.054 | 0.039 | |
| **Wound Infection/Complication** | 460 (14.9%) | 164 (2.7%) | 298 (12.2%) | | <0.001 | <0.001 | <0.001 | |
| **Major Reintervention of Treated Segment** | 139 (4.5%) | 289 (4.7%) | 95 (3.9%) | | 0.255 | 0.639 | 0.098 | |
| **Major Amputation (Transtibial or Proximal)** | 79 (2.6%) | 280 (4.6%) | 85 (3.5%) | | <0.001 | <0.001 | 0.025 | |
| **Death** | 69 (2.2%) | 168 (2.7%) | 69 (2.8%) | | 0.275 | 0.145 | 0.821 | |
| **Readmission** | 497 (18.5%) | 1,011 (19.7%) | 427 (19.8%) | | 0.38 | 0.189 | 0.979 | |
| **Unplanned Readmission** | 478 (17.9%) | 911 (18.1%) | 413 (19.2%) | | 0.447 | 0.814 | 0.271 | |
| **Related Readmission** | 381 (14.8%) | 524 (11.3%) | 322 (15.7%) | | <0.001 | <0.001 | <0.001 | |
| **Serious Complication** | 718 (23.3%) | 1,156 (18.9%) | 584 (24.0%) | | <0.001 | <0.001 | <0.001 | |
| **Any Complication** | 867 (28.1%) | 1,202 (19.6%) | 656 (27.0%) | | <0.001 | <0.001 | <0.001 | |
| **Cardiac Arrest** | 28 (0.9%) | 58 (0.9%) | 34 (1.4%) | | 0.131 | 0.849 | 0.069 | |
| **Myocardial Infarction** | 93 (3.0%) | 147 (2.4%) | 82 (3.4%) | | 0.03 | 0.082 | 0.013 | |
| **Cardiac Complication** | 112 (3.6%) | 188 (3.1%) | 108 (4.4%) | | 0.008 | 0.155 | 0.002 | |
| **Pneumonia** | 50 (1.6%) | 96 (1.6%) | 42 (1.7%) | | 0.874 | 0.852 | 0.604 | |
| **Deep Incisional SSI** | 65 (2.1%) | 29 (0.5%) | 55 (2.3%) | | <0.001 | <0.001 | <0.001 | |
| **Organ Space SSI** | 23 (0.7%) | 13 (0.2%) | 30 (1.2%) | | <0.001 | <0.001 | <0.001 | |
| **Superficial Incisional SSI** | 242 (7.8%) | 56 (0.9%) | 122 (5.0%) | | <0.001 | <0.001 | <0.001 | |
| **SSI** | 323 (10.5%) | 98 (1.6%) | 203 (8.3%) | | <0.001 | <0.001 | <0.001 | |
| **UTI** | 49 (1.6%) | 54 (0.9%) | 41 (1.7%) | | 0.001 | 0.002 | 0.001 | |
| **VTE** | 31 (1.0%) | 46 (0.8%) | 22 (0.9%) | | 0.436 | 0.208 | 0.475 | |
| **Acute Renal Failure** | 11 (0.4%) | 37 (0.6%) | 14 (0.6%) | | 0.288 | 0.119 | 0.873 | |
| **Progressive Renal Insufficiency** | 14 (0.5%) | 33 (0.5%) | 8 (0.3%) | | 0.433 | 0.587 | 0.203 | |
| **Renal Failure** | 25 (0.8%) | 70 (1.1%) | 22 (0.9%) | | 0.271 | 0.135 | 0.331 | |
| **Return to OR** | 467 (15.1%) | 788 (12.9%) | 363 (14.9%) | | 0.003 | 0.003 | 0.013 | |
| **Reoperation** | 468 (15.2%) | 788 (12.9%) | 363 (14.9%) | | 0.003 | 0.003 | 0.013 | |
| **Wound Distruption** | 48 (1.6%) | 22 (0.4%) | 38 (1.6%) | | <0.001 | <0.001 | <0.001 | |
| **DVT** | 28 (0.9%) | 39 (0.6%) | 20 (0.8%) | | 0.326 | 0.15 | 0.353 | |
| **PE** | 4 (0.1%) | 9 (0.1%) | 2 (0.1%) | | 0.752 | 0.833 | 0.45 | |
| **Unplanned Intubation** | 48 (1.6%) | 91 (1.5%) | 43 (1.8%) | | 0.643 | 0.799 | 0.348 | |
| **Failure to Wean** | 27 (0.9%) | 58 (0.9%) | 22 (0.9%) | | 0.938 | 0.73 | 0.848 | |
| **Stroke CVA** | 16 (0.5%) | 29 (0.5%) | 13 (0.5%) | | 0.922 | 0.772 | 0.72 | |
| **Septic Shock** | 30 (1.0%) | 43 (0.7%) | 30 (1.2%) | | 0.051 | 0.169 | 0.016 | |
| **Sepsis** | 70 (2.3%) | 116 (1.9%) | 67 (2.8%) | | 0.045 | 0.23 | 0.014 | |

**Supplemental Table S6:** Regression Analysis of 30-day outcomes, Femoral-Popliteal

| **Major Amputation (Transtibial or Proximal)** | | | | | |
| --- | --- | --- | --- | --- | --- |
|  | Odds Ratio | 95% lower bound | 95% upper bound | | p-value |
| Procedure Type |  |  |  | |  |
| ENDO | 1.000 |  |  | |  |
| OPEN-GSV | 0.586 | 0.430 | 0.800 | | 0.001 |
| OPEN-OTHER | 0.769 | 0.580 | 1.020 | | 0.069 |
| Age | 0.993 | 0.982 | 1.004 | | 0.198 |
| SEX |  |  |  | |  |
| female | 1.000 |  |  | |  |
| male | 1.133 | 0.907 | 1.415 | | 0.272 |
| BMI | 1.001 | 0.983 | 1.020 | | 0.873 |
| Race |  |  |  | |  |
| White | 1.000 |  |  | |  |
| Black or African American | 1.335 | 1.048 | 1.702 | | 0.020 |
| American Indian or Alaska Native | 2.274 | 0.778 | 6.652 | | 0.134 |
| Asian | 0.957 | 0.380 | 2.409 | | 0.925 |
| Native Hawaiian or Pacific Islander | 1.000 |  |  | |  |
| High Risk Factors, Physiologic | 0.961 | 0.711 | 1.298 | | 0.796 |
| High Risk Factors, Anatomic |  |  |  | |  |
| None | 1.000 |  |  | |  |
| Prior Bypass | 1.844 | 1.409 | 2.414 | | <0.001 |
| Prior Endovascular | 1.058 | 0.786 | 1.425 | | 0.710 |
| Pre-procedural Antiplatelet Medication | 0.786 | 0.594 | 1.041 | | 0.093 |
| Pre-procedural Medication-Statin | 0.708 | 0.554 | 0.905 | | 0.006 |
| Functional Health Status |  |  |  | |  |
| Independent | 1.000 |  |  | |  |
| Partially Dependent | 1.324 | 0.978 | 1.794 | | 0.070 |
| Totally Dependent | 2.608 | 1.406 | 4.838 | | 0.002 |
| DIABETES |  |  |  | |  |
| None | 1.000 |  |  | |  |
| Non-Insulin Dependent | 0.859 | 0.613 | 1.205 | | 0.380 |
| Insulin Dependent | 1.180 | 0.913 | 1.526 | | 0.206 |
| Current Smoker within 1 year | 0.833 | 0.646 | 1.074 | | 0.159 |
| Ascites | 6.835 | 2.137 | 21.860 | | 0.001 |
| CHF with prior 30 days | 0.851 | 0.519 | 1.397 | | 0.524 |
| Acute Renal Failure (Pre-Op) | 1.738 | 0.981 | 3.082 | | 0.058 |
| Currently on Dialysis | 1.844 | 1.305 | 2.607 | | 0.001 |
| Disseminated Cancer | 2.654 | 1.077 | 6.543 | | 0.034 |
| Open wound/wound infection | 1.046 | 0.834 | 1.313 | | 0.694 |
| Steroid use for chronic condition | 1.228 | 0.816 | 1.849 | | 0.325 |
| >10% loss body weight in last 6 months | 2.801 | 1.585 | 4.949 | | <0.001 |
| Preop Transfusion with 72 hrs prior to surgery | 1.703 | 0.994 | 2.916 | | 0.053 |
| Systemic Sepsis |  |  |  | |  |
| None | 1.000 |  |  | |  |
| SIRS | 2.510 | 1.779 | 3.542 | | <0.001 |
| Sepsis | 3.978 | 2.385 | 6.635 | | <0.001 |
| Septic Shock | collinear |  |  | |  |
| **Major Adverse Limb Event** | | | | | |
|  | Odds Ratio | 95% lower bound | 95% upper bound | p-value | |
| Procedure Type |  |  |  |  | |
| ENDO | 1.000 |  |  |  | |
| OPEN-GSV | 0.708 | 0.576 | 0.870 | 0.001 | |
| OPEN-OTHER | 0.692 | 0.562 | 0.854 | 0.001 | |
| Age | 0.994 | 0.985 | 1.002 | 0.127 | |
| SEX |  |  |  |  | |
| female | 1.000 |  |  |  | |
| male | 0.992 | 0.846 | 1.163 | 0.921 | |
| BMI | 1.003 | 0.990 | 1.016 | 0.654 | |
| Race |  |  |  |  | |
| White | 1.000 |  |  |  | |
| Black or African American | 1.120 | 0.934 | 1.344 | 0.221 | |
| American Indian or Alaska Native | 1.668 | 0.687 | 4.053 | 0.259 | |
| Asian | 0.958 | 0.495 | 1.852 | 0.897 | |
| Native Hawaiian or Pacific Islander | 1.000 |  |  |  | |
| High Risk Factors, Physiologic | 1.094 | 0.886 | 1.351 | 0.403 | |
| High Risk Factors, Anatomic |  |  |  |  | |
| None | 1.000 |  |  |  | |
| Prior Bypass | 1.872 | 1.541 | 2.273 | <0.001 | |
| Prior Endovascular | 1.275 | 1.040 | 1.564 | 0.019 | |
| Pre-procedural Antiplatelet Medication | 0.752 | 0.612 | 0.923 | 0.006 | |
| Pre-procedural Medication-Statin | 0.931 | 0.775 | 1.119 | 0.446 | |
| Functional Health Status |  |  |  |  | |
| Independent | 1.000 |  |  |  | |
| Partially Dependent | 1.279 | 1.018 | 1.606 | 0.034 | |
| Totally Dependent | 1.521 | 0.868 | 2.668 | 0.143 | |
| DIABETES |  |  |  |  | |
| None | 1.000 |  |  |  | |
| Non-Insulin Dependent | 0.827 | 0.654 | 1.045 | 0.112 | |
| Insulin Dependent | 1.038 | 0.860 | 1.254 | 0.695 | |
| Current Smoker within 1 year | 0.983 | 0.820 | 1.178 | 0.851 | |
| Ascites | 5.745 | 1.906 | 17.317 | 0.002 | |
| Acute Renal Failure (Pre-Op) | 1.452 | 0.873 | 2.414 | 0.150 | |
| Currently on Dialysis | 1.353 | 1.032 | 1.772 | 0.028 | |
| Disseminated Cancer | 1.584 | 0.699 | 3.589 | 0.271 | |
| Open wound/wound infection | 0.979 | 0.832 | 1.152 | 0.796 | |
| Steroid use for chronic condition | 1.194 | 0.879 | 1.621 | 0.256 | |
| >10% loss body weight in last 6 months | 2.364 | 1.469 | 3.805 | <0.001 | |
| Systemic Sepsis |  |  |  |  | |
| None | 1.000 |  |  |  | |
| SIRS | 1.972 | 1.485 | 2.620 | <0.001 | |
| Sepsis | 2.515 | 1.589 | 3.980 | <0.001 | |
| Septic Shock | 1.000 |  |  |  | |
| Hypertension requiring medications | 0.839 | 0.680 | 1.034 | 0.100 | |
| **Perioperative Death** | | | | | |
|  | Odds Ratio | 95% lower bound | 95% upper bound | p-value | |
| **Procedure Type** |  |  |  |  | |
| ENDO | 1.000 |  |  |  | |
| OPEN-GSV | 1.247 | 0.900 | 1.726 | 0.184 | |
| OPEN-Other | 1.245 | 0.912 | 1.699 | 0.169 | |
| **Age** | 1.051 | 1.036 | 1.067 | <0.001 | |
| **Gender** |  |  |  |  | |
| female | 1.000 |  |  |  | |
| male | 0.958 | 0.744 | 1.233 | 0.738 | |
| **BMI** | 1.000 | 0.978 | 1.022 | 0.976 | |
| **High Risk Factors, Physiologic** | 1.454 | 1.052 | 2.009 | 0.023 | |
| **High Risk Factors, Anatomic** |  |  |  |  | |
| None | 1.000 |  |  |  | |
| Prior Bypass | 0.958 | 0.668 | 1.374 | 0.816 | |
| Prior Endovascular Intervention | 0.926 | 0.664 | 1.292 | 0.652 | |
| **Pre-procedural Antiplatelet Medication** | 1.277 | 0.897 | 1.820 | 0.175 | |
| **Pre-procedural Medication-Statin** | 0.779 | 0.586 | 1.036 | 0.086 | |
| **Functional Health Status** |  |  |  |  | |
| Independent | 1.000 |  |  |  | |
| Partially Dependent | 1.165 | 0.845 | 1.607 | 0.351 | |
| Totally Dependent | 1.515 | 0.761 | 3.020 | 0.237 | |
| **Diabetes** |  |  |  |  | |
| None | 1.000 |  |  |  | |
| Non-Insulin Dependent | 1.022 | 0.715 | 1.460 | 0.906 | |
| Insulin Dependent | 1.080 | 0.794 | 1.468 | 0.625 | |
| **Current Smoker within 1 year** | 0.814 | 0.585 | 1.134 | 0.224 | |
| **Ascites** | 8.976 | 2.616 | 30.800 | <0.001 | |
| **CHF with prior 30 days** | 2.551 | 1.751 | 3.715 | <0.001 | |
| **Acute Renal Failure (Pre-Op)** | 0.893 | 0.452 | 1.763 | 0.744 | |
| **Currently on Dialysis** | 3.407 | 2.434 | 4.767 | <0.001 | |
| **Disseminated Cancer** | 8.040 | 3.763 | 17.176 | <0.001 | |
| **Open wound/wound infection** | 0.994 | 0.767 | 1.287 | 0.962 | |
| **Steroid use for chronic condition** | 1.480 | 0.969 | 2.260 | 0.070 | |
| **>10% loss body weight in last 6 months** | 1.560 | 0.768 | 3.170 | 0.219 | |
| **Preop Transfusion with 72 hrs prior to surgery** | 1.603 | 0.857 | 2.996 | 0.139 | |
| **Systemic Sepsis** |  |  |  |  | |
| None | 1.000 |  |  |  | |
| SIRS | 2.816 | 1.904 | 4.164 | <0.001 | |
| Sepsis | 2.608 | 1.287 | 5.286 | 0.008 | |
| Septic Shock | 10.809 | 1.622 | 72.018 | 0.014 | |
| **Hypertension requiring medications** | 0.929 | 0.635 | 1.361 | 0.707 | |
| **Dyspnea** |  |  |  |  | |
| No | 1.000 |  |  |  | |
| Moderate Exertion | 1.366 | 0.967 | 1.927 | 0.076 | |
| At Rest | 2.546 | 1.243 | 5.213 | 0.011 | |
| **Pre-procedural Medication-Beta Blocker** | 1.164 | 0.870 | 1.556 | 0.306 | |
| **History of Severe COPD** | 1.520 | 1.101 | 2.098 | 0.011 | |
| **MALE or Perioperative Death** | | | | | |
|  | Odds Ratio | 95% lower bound | 95% upper bound | p-value | |
| **Procedure Type** |  |  |  |  | |
| ENDO | 1.000 |  |  |  | |
| OPEN-GSV | 0.820 | 0.682 | 0.986 | 0.035 | |
| OPEN-Other | 0.778 | 0.646 | 0.936 | 0.008 | |
| **Age** | 1.003 | 0.996 | 1.011 | 0.376 | |
| **Gender** |  |  |  |  | |
| female | 1.000 |  |  |  | |
| male | 0.976 | 0.846 | 1.126 | 0.738 | |
| **BMI** | 1.002 | 0.990 | 1.014 | 0.727 | |
| **Race** |  |  |  |  | |
| White | 1.000 |  |  |  | |
| Black or African American | 0.998 | 0.844 | 1.181 | 0.982 | |
| American Indian or Alaska Native | 1.398 | 0.602 | 3.250 | 0.436 | |
| Asian | 0.862 | 0.479 | 1.554 | 0.622 | |
| Native Hawaiian or Pacific Islander | 0.625 | 0.080 | 4.882 | 0.654 | |
| **High Risk Factors, Physiologic** | 1.225 | 1.016 | 1.478 | 0.034 | |
| **High Risk Factors, Anatomic** |  |  |  |  | |
| None | 1.000 |  |  |  | |
| Prior Bypass | 1.663 | 1.391 | 1.989 | <0.001 | |
| Prior Endovascular Intervention | 1.212 | 1.010 | 1.456 | 0.039 | |
| **Pre-procedural Antiplatelet Medication** | 0.831 | 0.689 | 1.003 | 0.054 | |
| **Pre-procedural Medication-Statin** | 0.882 | 0.748 | 1.039 | 0.132 | |
| **Functional Health Status** |  |  |  |  | |
| Independent | 1.000 |  |  |  | |
| Partially Dependent | 1.247 | 1.021 | 1.524 | 0.031 | |
| Totally Dependent | 1.807 | 1.139 | 2.869 | 0.012 | |
| **Diabetes** |  |  |  |  | |
| None | 1.000 |  |  |  | |
| Non-Insulin Dependent | 0.874 | 0.710 | 1.075 | 0.203 | |
| Insulin Dependent | 1.045 | 0.883 | 1.237 | 0.605 | |
| **Current Smoker within 1 year** | 0.924 | 0.782 | 1.092 | 0.352 | |
| **Ascites** | 8.734 | 2.777 | 27.467 | <0.001 | |
| **CHF with prior 30 days** | 1.446 | 1.079 | 1.938 | 0.013 | |
| **Acute Renal Failure (Pre-Op)** | 1.251 | 0.791 | 1.977 | 0.338 | |
| **Currently on Dialysis** | 1.691 | 1.344 | 2.129 | <0.001 | |
| **Disseminated Cancer** | 3.208 | 1.720 | 5.983 | <0.001 | |
| **Steroid use for chronic condition** | 1.256 | 0.961 | 1.641 | 0.095 | |
| **>10% loss body weight in last 6 months** | 2.230 | 1.440 | 3.456 | <0.001 | |
| **Preop Transfusion with 72 hrs prior to surgery** | 1.412 | 0.935 | 2.133 | 0.101 | |
| **Systemic Sepsis** |  |  |  |  | |
| None | 1.000 |  |  |  | |
| SIRS | 2.287 | 1.779 | 2.939 | <0.001 | |
| Sepsis | 2.525 | 1.651 | 3.860 | <0.001 | |
| Septic Shock | 1.692 | 0.165 | 17.385 | 0.658 | |
| **Hypertension requiring medications** | 0.897 | 0.739 | 1.090 | 0.274 | |
| **Dyspnea** |  |  |  |  | |
| No | 1.000 |  |  |  | |
| Moderate Exertion | 1.044 | 0.839 | 1.300 | 0.698 | |
| At Rest | 1.638 | 0.934 | 2.872 | 0.085 | |
| **Major Reintervention of Treated Segment** | | | | | |
|  | Odds Ratio | 95% lower bound | 95% upper bound | p-value | |
| **Procedure Type** |  |  |  |  | |
| ENDO | 1.000 |  |  |  | |
| OPEN-GSV | 0.863 | 0.673 | 1.107 | 0.247 | |
| OPEN-Other | 0.763 | 0.585 | 0.995 | 0.046 | |
| **Age** | 0.995 | 0.985 | 1.006 | 0.395 | |
| **Gender** |  |  |  |  | |
| female | 1.000 |  |  |  | |
| male | 0.875 | 0.716 | 1.070 | 0.194 | |
| **BMI** | 1.004 | 0.987 | 1.021 | 0.649 | |
| **Race** |  |  |  |  | |
| White | 1.000 |  |  |  | |
| Black or African American | 0.927 | 0.729 | 1.181 | 0.541 | |
| American Indian or Alaska Native | 2.021 | 0.712 | 5.738 | 0.186 | |
| Asian | 0.981 | 0.426 | 2.256 | 0.964 | |
| Native Hawaiian or Pacific Islander | 1.000 |  |  |  | |
| **High Risk Factors, Physiologic** | 1.193 | 0.913 | 1.558 | 0.197 | |
| **High Risk Factors, Anatomic** |  |  |  |  | |
| None | 1.000 |  |  |  | |
| Prior Bypass | 1.842 | 1.443 | 2.351 | <0.001 | |
| Prior Endovascular Intervention | 1.396 | 1.082 | 1.802 | 0.010 | |
| **Pre-procedural Antiplatelet Medication** | 0.659 | 0.510 | 0.850 | 0.001 | |
| **Pre-procedural Medication-Statin** | 1.087 | 0.857 | 1.378 | 0.493 | |
| **Diabetes** |  |  |  |  | |
| None | 1.000 |  |  |  | |
| Non-Insulin Dependent | 0.807 | 0.603 | 1.078 | 0.146 | |
| Insulin Dependent | 0.905 | 0.710 | 1.155 | 0.424 | |
| **Current Smoker within 1 year** | 1.127 | 0.897 | 1.416 | 0.303 | |
| **Acute Renal Failure (Pre-Op)** | 0.717 | 0.281 | 1.827 | 0.485 | |
| **Currently on Dialysis** | 0.973 | 0.664 | 1.426 | 0.889 | |
| **Open wound/wound infection** | 0.890 | 0.724 | 1.094 | 0.269 | |
| **Systemic Sepsis** |  |  |  |  | |
| None | 1.000 |  |  |  | |
| SIRS | 1.651 | 1.114 | 2.445 | 0.012 | |
| Sepsis | 1.667 | 0.833 | 3.338 | 0.149 | |
| Septic Shock | 1.000 |  |  |  | |
| **Hypertension requiring medications** | 0.893 | 0.684 | 1.165 | 0.404 | |
| **Major Adverse Cardiovascular Event** | | | | | |
|  | Odds Ratio | 95% lower bound | 95% upper bound | p-value | |
| **Procedure Type** |  |  |  |  | |
| ENDO | 1.000 |  |  |  | |
| OPEN-GSV | 1.661 | 1.173 | 2.351 | 0.004 | |
| OPEN-Other | 1.631 | 1.161 | 2.290 | 0.005 | |
| **Age** | 1.012 | 0.996 | 1.028 | 0.149 | |
| **Gender** |  |  |  |  | |
| female | 1.000 |  |  |  | |
| male | 1.100 | 0.825 | 1.466 | 0.518 | |
| **BMI** | 0.990 | 0.966 | 1.015 | 0.449 | |
| **Race** |  |  |  |  | |
| White | 1.000 |  |  |  | |
| Black or African American | 0.712 | 0.494 | 1.026 | 0.068 | |
| American Indian or Alaska Native | 0.794 | 0.107 | 5.877 | 0.821 | |
| Asian | 1.091 | 0.393 | 3.026 | 0.867 | |
| Native Hawaiian or Pacific Islander | 1.000 |  |  |  | |
| **High Risk Factors, Physiologic** | 1.103 | 0.765 | 1.592 | 0.599 | |
| **High Risk Factors, Anatomic** |  |  |  |  | |
| None | 1.000 |  |  |  | |
| Prior Bypass | 1.102 | 0.764 | 1.589 | 0.602 | |
| Prior Endovascular Intervention | 1.037 | 0.721 | 1.492 | 0.844 | |
| **Pre-procedural Antiplatelet Medication** | 1.206 | 0.784 | 1.855 | 0.395 | |
| **Pre-procedural Medication-Statin** | 1.138 | 0.798 | 1.623 | 0.475 | |
| **Diabetes** |  |  |  |  | |
| None | 1.000 |  |  |  | |
| Non-Insulin Dependent | 1.402 | 0.951 | 2.067 | 0.088 | |
| Insulin Dependent | 1.484 | 1.057 | 2.084 | 0.023 | |
| **Current Smoker within 1 year** | 0.810 | 0.578 | 1.134 | 0.219 | |
| **CHF with prior 30 days** | 1.113 | 0.611 | 2.027 | 0.726 | |
| **Currently on Dialysis** | 1.550 | 0.984 | 2.442 | 0.059 | |
| **Disseminated Cancer** | 3.324 | 1.154 | 9.572 | 0.026 | |
| **>10% loss body weight in last 6 months** | 1.194 | 0.421 | 3.390 | 0.739 | |
| **Systemic Sepsis** |  |  |  |  | |
| None | 1.000 |  |  |  | |
| SIRS | 1.506 | 0.865 | 2.622 | 0.147 | |
| Sepsis | 1.073 | 0.334 | 3.452 | 0.906 | |
| Septic Shock | 10.100 | 1.006 | 101.449 | 0.049 | |
| **Hypertension requiring medications** | 1.676 | 0.992 | 2.833 | 0.054 | |
| **Dyspnea** |  |  |  |  | |
| No | 1.000 |  |  |  | |
| Moderate Exertion | 1.216 | 0.802 | 1.844 | 0.357 | |
| At Rest | 1.399 | 0.421 | 4.646 | 0.584 | |
| **Pre-procedural Medication-Beta Blocker** | 1.254 | 0.904 | 1.739 | 0.175 | |
| **Bleeding Disorder** | 1.215 | 0.905 | 1.633 | 0.195 | |

**Supplemental Table S7:** Inverse-propensity weighted with regression adjustment analysis, Femoral-Popliteal

| **ENDO vs. OPEN-GSV** | | | | |
| --- | --- | --- | --- | --- |
| **Variable** | **Risk Ratio** | **95% lower bound** | **95% upper bound** | **p-value** |
| Major Amputation | 1.50 | 1.07 | 2.10 | 0.019 |
| MALE | 1.33 | 1.07 | 1.65 | 0.010 |
| POD | 0.77 | 0.53 | 1.12 | 0.17 |
| MALE or POD | 1.16 | 0.97 | 1.40 | 0.11 |
| Major Reintervention | 1.14 | 0.87 | 1.50 | 0.34 |
| MACE | 0.64 | 0.46 | 0.90 | 0.011 |
| **ENDO vs. OPEN-Other** | | | | |
| **Variable** | **Risk Ratio** | **95% lower bound** | **95% upper bound** | **p-value** |
| Major Amputation | 1.29 | 0.96 | 1.74 | 0.09 |
| MALE | 1.45 | 1.17 | 1.79 | <0.001 |
| POD | 0.79 | 0.57 | 1.09 | 0.15 |
| MALE or POD | 1.26 | 1.06 | 1.51 | 0.011 |
| Major Reintervention | 1.31 | 0.99 | 1.75 | 0.059 |
| MACE | 0.60 | 0.43 | 0.84 | 0.003 |

**Supplemental Table S8:** Patient Demographics**,** Comparison of OPEN-GSV vs. ENDO vs. OPEN-Other, Femoral-Tibial

|  | Procedure Type | | | | P-value | | | |
| --- | --- | --- | --- | --- | --- | --- | --- | --- |
|  | **OPEN-GSV**  **(n=2,658)** | **ENDO**  **(n=2,769)** | **OPEN-OTHER**  **(n=1,498)** | **Overall** | | **ENDO vs. OPEN-GSV** | **ENDO vs. OPEN-Other** |  |
| **Age** | 68.5 (11.811) | 69.4 (12.2) | 70.0 (10.8) | <0.001 | | 0.006 | 0.08 |  |
| **BMI** | 27.988 (6.028) | 28.405 (6.298) | 27.327 (5.945) | <0.001 | | 0.015 | <0.001 |  |
| **Female Sex** | 765 (28.8%) | 887 (32.0%) | 523 (34.9%) | <0.001 | | 0.009 | 0.056 |  |
| **RACE** |  |  |  |  | |  |  |  |
| American Indian or Alaska Native | 8 (0.4%) | 14 (0.6%) | 3 (0.2%) | <0.001 | | <0.001 | <0.001 |  |
| Asian | 19 (1.0%) | 63 (2.6%) | 8 (0.6%) |  | |  |  |  |
| Black or African American | 476 (23.8%) | 717 (29.6%) | 312 (25.2%) |  | |  |  |  |
| Native Hawaiian or Pacific Islander | 2 (0.1%) | 6 (0.2%) | 2 (0.2%) |  | |  |  |  |
| White | 1,494 (74.7%) | 1,620 (66.9%) | 915 (73.8%) |  | |  |  |  |
| Functional Health Status |  |  |  |  | |  |  |  |
| Independent | 2,448 (92.6%) | 2,277 (82.7%) | 1,329 (89.2%) | <0.001 | | <0.001 | <0.001 |  |
| Partially Dependent | 185 (7.0%) | 427 (15.5%) | 145 (9.7%) |  | |  |  |  |
| Totally Dependent | 10 (0.4%) | 48 (1.7%) | 16 (1.1%) |  | |  |  |  |
| **DIABETES** |  |  |  |  | |  |  |  |
| None | 1,329 (50.0%) | 890 (32.1%) | 733 (48.9%) | <0.001 | | <0.001 | <0.001 |  |
| Non-Insulin Dependent | 530 (19.9%) | 551 (19.9%) | 299 (20.0%) |  | |  |  |  |
| Insulin Dependent | 799 (30.1%) | 1,328 (48.0%) | 466 (31.1%) |  | |  |  |  |
| **Current Smoker within 1 year** | 1,038 (39.1%) | 457 (16.5%) | 540 (36.0%) | <0.001 | | <0.001 | <0.001 |  |
| **DYSPNEA** |  |  |  | 0.245 | | 0.102 | 0.978 |  |
| No | 2,404 (90.4%) | 2,479 (89.5%) | 1,342 (89.6%) |  | |  |  |  |
| Moderate Exertion | 246 (9.3%) | 271 (9.8%) | 145 (9.7%) |  | |  |  |  |
| At Rest | 8 (0.3%) | 19 (0.7%) | 11 (0.7%) |  | |  |  |  |
| Ventilator Dependent | 6 (0.2%) | 4 (0.1%) | 6 (0.4%) | 0.25 | | 0.485 | 0.099 |  |
| **History of Severe COPD** | 275 (10.3%) | 185 (6.7%) | 163 (10.9%) | <0.001 | | <0.001 | <0.001 |  |
| **Ascites** | 3 (0.1%) | 7 (0.3%) | 1 (0.1%) | 0.26 | | 0.23 | 0.18 |  |
| **CHF with prior 30 days** | 90 (3.4%) | 137 (4.9%) | 63 (4.2%) | 0.016 | | 0.004 | 0.274 |  |
| **Hypertension requiring medications** | 2,122 (79.8%) | 2,344 (84.7%) | 1,287 (85.9%) | <0.001 | | <0.001 | 0.269 |  |
| **Acute Renal Failure (Pre-Op)** | 34 (1.3%) | 61 (2.2%) | 24 (1.6%) | 0.03 | | 0.009 | 0.18 |  |
| **Currently on Dialysis** | 167 (6.3%) | 487 (17.6%) | 116 (7.7%) | <0.001 | | <0.001 | <0.001 |  |
| **Disseminated Cancer** | 13 (0.5%) | 11 (0.4%) | 11 (0.7%) | 0.33 | | 0.61 | 0.142 |  |
| **Open wound/wound infection** | 1,308 (49.2%) | 1,684 (60.8%) | 703 (46.9%) | <0.001 | | <0.001 | <0.001 |  |
| **Steroid use for chronic condition** | 129 (4.9%) | 231 (8.3%) | 75 (5.0%) | <0.001 | | <0.001 | <0.001 |  |
| **>10% loss body weight in last 6 months** | 27 (1.0%) | 48 (1.7%) | 19 (1.3%) | 0.07 | | 0.024 | 0.243 |  |
| **Bleeding Disorder** | 524 (23.2%) | 654 (29.6%) | 350 (27.6%) | <0.001 | | <0.001 | 0.215 |  |
| **Preop Transfusion with 72 hrs prior to surgery** | 60 (2.3%) | 48 (1.7%) | 37 (2.5%) | 0.209 | | 0.167 | 0.1 |  |
| **Systemic Sepsis** |  |  |  |  | |  |  |  |
| None | 2,533 (95.3%) | 2,499 (90.2%) | 1,420 (94.8%) | <0.001 | | <0.001 | <0.001 |  |
| SIRS | 101 (3.8%) | 192 (6.9%) | 67 (4.5%) |  | |  |  |  |
| Sepsis | 20 (0.8%) | 74 (2.7%) | 11 (0.7%) |  | |  |  |  |
| Septic Shock | 4 (0.2%) | 4 (0.1%) | 0 (0.0%) |  | |  |  |  |

**Supplemental Table S9:** Procedural Data, Comparison of OPEN-GSV vs. ENDO vs. OPEN-Other, Femoral-Tibial

|  | Procedure Type | | | | P-value | | |  |
| --- | --- | --- | --- | --- | --- | --- | --- | --- |
|  | **OPEN-GSV**  **(n=2,658)** | **ENDO**  **(n=2,769)** | **OPEN-OTHER**  **(n=1,498)** | **Overall** | | **ENDO vs. OPEN-GSV** | **ENDO vs. OPEN-Other** | |
| **Symptomatology** |  |  |  |  | |  |  | |
| Critical limb ischemia: rest pain | 1,011 (38.0%) | 494 (17.8%) | 600 (40.1%) | <0.001 | | <0.001 | <0.001 | |
| Critical limb ischemia: tissue loss | 1,647 (62.0%) | 2,275 (82.2%) | 898 (59.9%) |  | |  |  | |
| **High Risk Factors, Physiologic** | 670 (25.3%) | 1,131 (41.0%) | 435 (29.1%) | <0.001 | | <0.001 | <0.001 | |
| **High Risk Factors, Anatomic** |  |  |  |  | |  |  | |
| None | 1,641 (61.7%) | 2,007 (72.5%) | 648 (43.3%) | <0.001 | | <0.001 | <0.001 | |
| Prior Bypass | 485 (18.2%) | 296 (10.7%) | 602 (40.2%) |  | |  |  | |
| Prior Endovascular | 532 (20.0%) | 466 (16.8%) | 248 (16.6%) |  | |  |  | |
| **Pre-procedural Antiplatelet Medication** | 2,096 (79.1%) | 2,210 (80.1%) | 1,283 (85.9%) | <0.001 | | 0.344 | <0.001 | |
| **Pre-procedural Medication-Statin** | 1,844 (69.7%) | 1,926 (69.9%) | 1,123 (75.3%) | <0.001 | | 0.861 | <0.001 | |
| **Pre-procedural Medication-Beta Blocker** | 1,490 (56.3%) | 1,708 (62.0%) | 946 (63.6%) | <0.001 | | <0.001 | 0.297 | |
| **Overall Procedure** |  |  |  |  | |  |  | |
| Angioplasty | - | 1,885 (68.1%) | - |  | |  |  | |
| Atherectomy | - | 647 (23.4%) | - |  | |  |  | |
| Bypass | 2,658 (100.0%) | - | 1,498 (100.0%) |  | |  |  | |
| Stenting | - | 237 (8.6%) | - |  | |  |  | |
| **ASACLAS** |  |  |  |  | |  |  | |
| 1-No Disturb | 1 (0.0%) | 2 (0.1%) | 0 (0.0%) | <0.001 | | <0.001 | <0.001 | |
| 2-Mild Disturb | 82 (3.1%) | 168 (6.4%) | 19 (1.3%) |  | |  |  | |
| 3-Severe Disturb | 1,787 (67.3%) | 1,682 (63.9%) | 982 (65.6%) |  | |  |  | |
| 4-Life Threat | 780 (29.4%) | 778 (29.5%) | 495 (33.1%) |  | |  |  | |
| 5-Moribund | 5 (0.2%) | 3 (0.1%) | 1 (0.1%) |  | |  |  | |
| **ANESTHES** |  |  |  |  | |  |  | |
| None | 0 (0.0%) | 8 (0.3%) | 1 (0.1%) | <0.001 | | <0.001 | <0.001 | |
| Epidural | 17 (0.6%) | 0 (0.0%) | 3 (0.2%) |  | |  |  | |
| General | 2,449 (92.2%) | 880 (31.8%) | 1,450 (96.8%) |  | |  |  | |
| Local | 0 (0.0%) | 23 (0.8%) | 0 (0.0%) |  | |  |  | |
| MAC/IV Sedation | 50 (1.9%) | 1,818 (65.7%) | 11 (0.7%) |  | |  |  | |
| Other | 3 (0.1%) | 6 (0.2%) | 2 (0.1%) |  | |  |  | |
| Regional | 5 (0.2%) | 21 (0.8%) | 4 (0.3%) |  | |  |  | |
| Spinal | 133 (5.0%) | 11 (0.4%) | 27 (1.8%) |  | |  |  | |
| **Elective Surgery** | 1,349 (50.8%) | 1,256 (45.4%) | 696 (46.5%) | <0.001 | | <0.001 | 0.503 | |
| **Operative Time** | 268.467 (113.289) | 106.936 (66.548) | 257.682 (116.218) | <0.001 | | <0.001 | <0.001 | |

**Supplemental Table S10:** 30-day outcomes, Comparison of OPEN-GSV vs. ENDO vs. OPEN-Other, Femoral-Tibial

|  | Procedure Type | | | P-value | | |
| --- | --- | --- | --- | --- | --- | --- |
|  | **OPEN-GSV**  **(n=2,658)** | **ENDO**  **(n=2,769)** | **OPEN-OTHER**  **(n=1,498)** | **Overall** | **ENDO vs. OPEN-GSV** | **ENDO vs. OPEN-Other** |
| **Total Length of Stay, in days** | 10.196 (8.300) | 6.772 (8.739) | 10.375 (8.160) | <0.001 | <0.001 | <0.001 |
| **DISCHARGE DESTINATION** |  |  |  |  |  |  |
| Home | 1,631 (62.0%) | 2,134 (77.3%) | 823 (55.3%) | <0.001 | <0.001 | <0.001 |
| Against Medical Advice (AMA) | 4 (0.2%) | 7 (0.3%) | 2 (0.1%) |  |  |  |
| Expired | 28 (1.1%) | 27 (1.0%) | 28 (1.9%) |  |  |  |
| Hospice | 3 (0.1%) | 11 (0.4%) | 3 (0.2%) |  |  |  |
| Rehab | 407 (15.5%) | 171 (6.2%) | 243 (16.3%) |  |  |  |
| Separate Acute Care | 46 (1.7%) | 31 (1.1%) | 32 (2.1%) |  |  |  |
| Skilled Care, Not Home | 502 (19.1%) | 377 (13.7%) | 354 (23.8%) |  |  |  |
| Unskilled Facility Not Home | 10 (0.4%) | 2 (0.1%) | 4 (0.3%) |  |  |  |
| **Non-Routine Discharge** | 999 (38.0%) | 608 (22.2%) | 647 (44.0%) | <0.001 | <0.001 | <0.001 |
| **Untreated Loss of Patency** | 66 (2.5%) | 42 (1.5%) | 50 (3.3%) | <0.001 | 0.011 | <0.001 |
| **Bleeding Requiring Transfusion or Secondary Procedure** | 538 (20.2%) | 203 (7.3%) | 364 (24.3%) | <0.001 | <0.001 | <0.001 |
| **Myocardial Infarction or Stroke** | 121 (4.6%) | 42 (1.5%) | 78 (5.2%) | <0.001 | <0.001 | <0.001 |
| **Wound Infection/Complication** | 398 (15.0%) | 60 (2.2%) | 220 (14.7%) | <0.001 | <0.001 | <0.001 |
| **Major Reintervention of Treated Segment** | 163 (6.1%) | 106 (3.8%) | 125 (8.3%) | <0.001 | <0.001 | <0.001 |
| **Major Amputation (Transtibial or Proximal** | 111 (4.2%) | 147 (5.3%) | 113 (7.5%) | <0.001 | 0.05 | 0.004 |
| **Death** | 60 (2.3%) | 76 (2.7%) | 44 (2.9%) | 0.344 | 0.251 | 0.716 |
| **Readmission** | 485 (20.8%) | 479 (20.8%) | 290 (22.3%) | 0.53 | 0.995 | 0.308 |
| **Unplanned Readmission** | 463 (20.1%) | 447 (19.7%) | 274 (21.3%) | 0.513 | 0.755 | 0.255 |
| **Related Readmission** | 366 (16.6%) | 244 (11.8%) | 216 (17.6%) | <0.001 | <0.001 | <0.001 |
| **Serious Complication** | 766 (28.8%) | 534 (19.3%) | 478 (31.9%) | <0.001 | <0.001 | <0.001 |
| **Any Complication** | 880 (33.1%) | 543 (19.6%) | 525 (35.0%) | <0.001 | <0.001 | <0.001 |
| **Cardiac Arrest** | 31 (1.2%) | 16 (0.6%) | 19 (1.3%) | 0.03 | 0.019 | 0.017 |
| **Myocardial Infarction** | 115 (4.3%) | 45 (1.6%) | 76 (5.1%) | <0.001 | <0.001 | <0.001 |
| **Cardiac Complication** | 137 (5.2%) | 57 (2.1%) | 92 (6.1%) | <0.001 | <0.001 | <0.001 |
| **Pneumonia** | 48 (1.8%) | 36 (1.3%) | 24 (1.6%) | 0.319 | 0.131 | 0.424 |
| **Deep Incisional SSI** | 71 (2.7%) | 6 (0.2%) | 37 (2.5%) | <0.001 | <0.001 | <0.001 |
| **Organ Space SSI** | 23 (0.9%) | 15 (0.5%) | 25 (1.7%) | 0.001 | 0.153 | <0.001 |
| **Superficial Incisional SSI** | 193 (7.3%) | 16 (0.6%) | 80 (5.3%) | <0.001 | <0.001 | <0.001 |
| **SSI** | 283 (10.6%) | 37 (1.3%) | 139 (9.3%) | <0.001 | <0.001 | <0.001 |
| **UTI** | 40 (1.5%) | 19 (0.7%) | 37 (2.5%) | <0.001 | 0.004 | <0.001 |
| **VTE** | 33 (1.2%) | 16 (0.6%) | 14 (0.9%) | 0.036 | 0.01 | 0.183 |
| **Acute Renal Failure** | 21 (0.8%) | 17 (0.6%) | 5 (0.3%) | 0.198 | 0.437 | 0.223 |
| **Progressive Renal Insufficiency** | 18 (0.7%) | 14 (0.5%) | 11 (0.7%) | 0.593 | 0.409 | 0.35 |
| **Renal Failure** | 39 (1.5%) | 30 (1.1%) | 16 (1.1%) | 0.359 | 0.207 | 0.963 |
| **Return to OR** | 532 (20.0%) | 388 (14.0%) | 344 (23.0%) | <0.001 | <0.001 | <0.001 |
| **Reoperation** | 532 (20.0%) | 388 (14.0%) | 344 (23.0%) | <0.001 | <0.001 | <0.001 |
| **Wound Distruption** | 49 (1.8%) | 2 (0.1%) | 29 (1.9%) | <0.001 | <0.001 | <0.001 |
| **DVT** | 20 (0.8%) | 12 (0.4%) | 12 (0.8%) | 0.221 | 0.125 | 0.125 |
| **PE** | 15 (0.6%) | 4 (0.1%) | 3 (0.2%) | 0.015 | 0.009 | 0.667 |
| **Unplanned Intubation** | 41 (1.5%) | 35 (1.3%) | 29 (1.9%) | 0.228 | 0.383 | 0.085 |
| **Failure to Wean** | 31 (1.2%) | 10 (0.4%) | 18 (1.2%) | 0.001 | <0.001 | 0.001 |
| **Stroke CVA** | 21 (0.8%) | 9 (0.3%) | 15 (1.0%) | 0.017 | 0.021 | 0.005 |
| **Septic Shock** | 33 (1.2%) | 23 (0.8%) | 18 (1.2%) | 0.289 | 0.134 | 0.236 |
| **Sepsis** | 53 (2.0%) | 72 (2.6%) | 38 (2.5%) | 0.294 | 0.137 | 0.901 |

**Supplemental Table S11:** Regression Analysis of 30-day outcomes, Femoral-Tibial

| **Major Amputation (Transtibial or Proximal)** | | | | | |
| --- | --- | --- | --- | --- | --- |
|  | Odds Ratio | 95% lower bound | 95% upper bound | | p-value |
| Procedure Type |  |  |  | |  |
| ENDO | 1.000 |  |  | |  |
| OPEN-GSV | 1.045 | 0.769 | 1.420 | | 0.780 |
| OPEN-OTHER | 1.623 | 1.176 | 2.242 | | 0.003 |
| Age | 0.989 | 0.978 | 1.001 | | 0.083 |
| SEX |  |  |  | |  |
| female | 1.000 |  |  | |  |
| male | 0.939 | 0.723 | 1.219 | | 0.637 |
| BMI | 0.999 | 0.979 | 1.020 | | 0.937 |
| Race |  |  |  | |  |
| White | 1.000 |  |  | |  |
| Black or African American | 1.389 | 1.066 | 1.809 | | 0.015 |
| American Indian or Alaska Native | 0.811 | 0.107 | 6.131 | | 0.839 |
| Asian | 0.473 | 0.113 | 1.977 | | 0.305 |
| Native Hawaiian or Pacific Islander | 0.974 | 0.109 | 8.723 | | 0.981 |
| High Risk Factors, Physiologic | 1.185 | 0.854 | 1.645 | | 0.309 |
| High Risk Factors, Anatomic |  |  |  | |  |
| None | 1.000 |  |  | |  |
| Prior Bypass | 1.615 | 1.188 | 2.197 | | 0.002 |
| Prior Endovascular | 0.920 | 0.643 | 1.318 | | 0.651 |
| Pre-procedural Antiplatelet Medication | 0.688 | 0.510 | 0.929 | | 0.015 |
| Pre-procedural Medication-Statin | 0.807 | 0.614 | 1.061 | | 0.124 |
| Functional Health Status |  |  |  | |  |
| Independent | 1.000 |  |  | |  |
| Partially Dependent | 1.112 | 0.769 | 1.606 | | 0.574 |
| Totally Dependent | 0.243 | 0.033 | 1.795 | | 0.165 |
| DIABETES |  |  |  | |  |
| None | 1.000 |  |  | |  |
| Non-Insulin Dependent | 0.904 | 0.635 | 1.287 | | 0.577 |
| Insulin Dependent | 0.934 | 0.698 | 1.249 | | 0.644 |
| Current Smoker within 1 year | 0.678 | 0.497 | 0.925 | | 0.014 |
| Ascites | 0.691 | 0.072 | 6.641 | | 0.749 |
| CHF with prior 30 days | 1.340 | 0.798 | 2.250 | | 0.268 |
| Acute Renal Failure (Pre-Op) | 1.145 | 0.537 | 2.445 | | 0.725 |
| Currently on Dialysis | 1.701 | 1.150 | 2.516 | | 0.008 |
| Disseminated Cancer | 0.520 | 0.066 | 4.111 | | 0.536 |
| Open wound/wound infection | 0.801 | 0.622 | 1.033 | | 0.087 |
| Steroid use for chronic condition | 1.644 | 1.085 | 2.490 | | 0.019 |
| >10% loss body weight in last 6 months | 1.719 | 0.783 | 3.775 | | 0.177 |
| Preop Transfusion with 72 hrs prior to surgery | 0.418 | 0.149 | 1.171 | | 0.097 |
| Systemic Sepsis |  |  |  | |  |
| None | 1.000 |  |  | |  |
| SIRS | 2.895 | 1.972 | 4.250 | | <0.001 |
| Sepsis | 6.168 | 3.528 | 10.784 | | <0.001 |
| Septic Shock | 5.021 | 0.570 | 44.261 | | 0.146 |
| **Major Adverse Limb Event** | | | | | |
|  | Odds Ratio | 95% lower bound | 95% upper bound | p-value | |
| Procedure Type |  |  |  |  | |
| ENDO | 1.000 |  |  |  | |
| OPEN-GSV | 1.205 | 0.960 | 1.513 | 0.108 | |
| OPEN-OTHER | 1.688 | 1.322 | 2.155 | <0.001 | |
| Age | 0.992 | 0.983 | 1.002 | 0.105 | |
| SEX |  |  |  |  | |
| female | 1.000 |  |  |  | |
| male | 1.020 | 0.837 | 1.244 | 0.841 | |
| BMI | 1.013 | 0.998 | 1.028 | 0.084 | |
| Race |  |  |  |  | |
| White | 1.000 |  |  |  | |
| Black or African American | 1.231 | 1.003 | 1.512 | 0.047 | |
| American Indian or Alaska Native | 0.412 | 0.055 | 3.089 | 0.389 | |
| Asian | 0.991 | 0.447 | 2.196 | 0.982 | |
| Native Hawaiian or Pacific Islander | 0.712 | 0.084 | 6.045 | 0.756 | |
| High Risk Factors, Physiologic | 1.084 | 0.848 | 1.386 | 0.521 | |
| High Risk Factors, Anatomic |  |  |  |  | |
| None | 1.000 |  |  |  | |
| Prior Bypass | 1.772 | 1.412 | 2.224 | <0.001 | |
| Prior Endovascular | 1.100 | 0.852 | 1.420 | 0.465 | |
| Pre-procedural Antiplatelet Medication | 0.816 | 0.642 | 1.036 | 0.095 | |
| Pre-procedural Medication-Statin | 0.907 | 0.734 | 1.120 | 0.365 | |
| Functional Health Status |  |  |  |  | |
| Independent | 1.000 |  |  |  | |
| Partially Dependent | 1.318 | 1.001 | 1.736 | 0.049 | |
| Totally Dependent | 0.496 | 0.152 | 1.613 | 0.244 | |
| DIABETES |  |  |  |  | |
| None | 1.000 |  |  |  | |
| Non-Insulin Dependent | 0.979 | 0.755 | 1.269 | 0.873 | |
| Insulin Dependent | 0.990 | 0.794 | 1.235 | 0.929 | |
| Current Smoker within 1 year | 0.901 | 0.722 | 1.124 | 0.355 | |
| Ascites | 0.632 | 0.073 | 5.506 | 0.678 | |
| Acute Renal Failure (Pre-Op) | 1.262 | 0.685 | 2.326 | 0.456 | |
| Currently on Dialysis | 1.337 | 0.971 | 1.841 | 0.075 | |
| Disseminated Cancer | 0.634 | 0.143 | 2.808 | 0.548 | |
| Open wound/wound infection | 0.799 | 0.661 | 0.965 | 0.020 | |
| Steroid use for chronic condition | 1.636 | 1.181 | 2.266 | 0.003 | |
| >10% loss body weight in last 6 months | 1.232 | 0.618 | 2.457 | 0.554 | |
| Systemic Sepsis |  |  |  |  | |
| None | 1.000 |  |  |  | |
| SIRS | 2.197 | 1.589 | 3.037 | <0.001 | |
| Sepsis | 3.504 | 2.085 | 5.890 | <0.001 | |
| Septic Shock | 5.558 | 1.051 | 29.406 | 0.044 | |
| Hypertension requiring medications | 0.986 | 0.765 | 1.271 | 0.915 | |
| **Perioperative Death** | | | | | |
|  | Odds Ratio | 95% lower bound | 95% upper bound | p-value | |
| **Procedure Type** |  |  |  |  | |
| ENDO | 1.000 |  |  |  | |
| OPEN-GSV | 1.173 | 0.785 | 1.753 | 0.437 | |
| OPEN-Other | 1.496 | 0.960 | 2.331 | 0.075 | |
| **Age** | 1.046 | 1.026 | 1.067 | <0.001 | |
| **Gender** |  |  |  |  | |
| female | 1.000 |  |  |  | |
| male | 0.958 | 0.675 | 1.359 | 0.809 | |
| **BMI** | 0.991 | 0.963 | 1.020 | 0.541 | |
| **High Risk Factors, Physiologic** | 1.671 | 1.084 | 2.575 | 0.020 | |
| **High Risk Factors, Anatomic** |  |  |  |  | |
| None | 1.000 |  |  |  | |
| Prior Bypass | 0.605 | 0.351 | 1.044 | 0.071 | |
| Prior Endovascular Intervention | 1.120 | 0.739 | 1.698 | 0.594 | |
| **Pre-procedural Antiplatelet Medication** | 1.053 | 0.677 | 1.636 | 0.820 | |
| **Pre-procedural Medication-Statin** | 0.817 | 0.561 | 1.189 | 0.291 | |
| **Functional Health Status** |  |  |  |  | |
| Independent | 1.000 |  |  |  | |
| Partially Dependent | 0.963 | 0.603 | 1.539 | 0.875 | |
| Totally Dependent | 3.523 | 1.536 | 8.081 | 0.003 | |
| **Diabetes** |  |  |  |  | |
| None | 1.000 |  |  |  | |
| Non-Insulin Dependent | 0.858 | 0.534 | 1.380 | 0.529 | |
| Insulin Dependent | 1.023 | 0.684 | 1.529 | 0.912 | |
| **Current Smoker within 1 year** | 0.889 | 0.548 | 1.445 | 0.636 | |
| **Ascites** | 5.700 | 1.153 | 28.180 | 0.033 | |
| **CHF with prior 30 days** | 2.229 | 1.320 | 3.766 | 0.003 | |
| **Acute Renal Failure (Pre-Op)** | 0.692 | 0.259 | 1.851 | 0.463 | |
| **Currently on Dialysis** | 2.576 | 1.618 | 4.100 | <0.001 | |
| **Disseminated Cancer** | 3.952 | 1.114 | 14.017 | 0.033 | |
| **Open wound/wound infection** | 0.813 | 0.577 | 1.144 | 0.234 | |
| **Steroid use for chronic condition** | 1.240 | 0.673 | 2.287 | 0.490 | |
| **>10% loss body weight in last 6 months** | 3.489 | 1.555 | 7.827 | 0.002 | |
| **Preop Transfusion with 72 hrs prior to surgery** | 0.996 | 0.401 | 2.474 | 0.993 | |
| **Systemic Sepsis** |  |  |  |  | |
| None | 1.000 |  |  |  | |
| SIRS | 2.849 | 1.735 | 4.678 | <0.001 | |
| Sepsis | 7.375 | 3.627 | 14.996 | <0.001 | |
| Septic Shock | 21.644 | 3.345 | 140.046 | 0.001 | |
| **Hypertension requiring medications** | 0.810 | 0.498 | 1.320 | 0.399 | |
| **Dyspnea** |  |  |  |  | |
| No | 1.000 |  |  |  | |
| Moderate Exertion | 1.379 | 0.852 | 2.232 | 0.190 | |
| At Rest | 1.853 | 0.411 | 8.345 | 0.422 | |
| **Pre-procedural Medication-Beta Blocker** | 1.298 | 0.883 | 1.906 | 0.184 | |
| **History of Severe COPD** | 1.025 | 0.586 | 1.791 | 0.932 | |
| **MALE or Perioperative Death** | | | | | |
|  | Odds Ratio | 95% lower bound | 95% upper bound | p-value | |
| **Procedure Type** |  |  |  |  | |
| ENDO | 1.000 |  |  |  | |
| OPEN-GSV | 1.155 | 0.937 | 1.423 | 0.177 | |
| OPEN-Other | 1.614 | 1.288 | 2.022 | <0.001 | |
| **Age** | 0.999 | 0.991 | 1.008 | 0.888 | |
| **Gender** |  |  |  |  | |
| female | 1.000 |  |  |  | |
| male | 0.977 | 0.814 | 1.173 | 0.803 | |
| **BMI** | 1.010 | 0.996 | 1.024 | 0.174 | |
| **Race** |  |  |  |  | |
| White | 1.000 |  |  |  | |
| Black or African American | 1.128 | 0.931 | 1.367 | 0.219 | |
| American Indian or Alaska Native | 0.329 | 0.044 | 2.460 | 0.279 | |
| Asian | 0.727 | 0.328 | 1.608 | 0.431 | |
| Native Hawaiian or Pacific Islander | 0.555 | 0.066 | 4.659 | 0.588 | |
| **High Risk Factors, Physiologic** | 1.218 | 0.972 | 1.526 | 0.086 | |
| **High Risk Factors, Anatomic** |  |  |  |  | |
| None | 1.000 |  |  |  | |
| Prior Bypass | 1.518 | 1.223 | 1.884 | <0.001 | |
| Prior Endovascular Intervention | 1.098 | 0.871 | 1.385 | 0.427 | |
| **Pre-procedural Antiplatelet Medication** | 0.837 | 0.670 | 1.046 | 0.118 | |
| **Pre-procedural Medication-Statin** | 0.918 | 0.755 | 1.116 | 0.391 | |
| **Functional Health Status** |  |  |  |  | |
| Independent | 1.000 |  |  |  | |
| Partially Dependent | 1.203 | 0.934 | 1.550 | 0.152 | |
| Totally Dependent | 1.341 | 0.661 | 2.720 | 0.416 | |
| **Diabetes** |  |  |  |  | |
| None | 1.000 |  |  |  | |
| Non-Insulin Dependent | 0.898 | 0.706 | 1.143 | 0.381 | |
| Insulin Dependent | 0.927 | 0.758 | 1.134 | 0.463 | |
| **Current Smoker within 1 year** | 0.901 | 0.730 | 1.111 | 0.329 | |
| **Ascites** | 3.006 | 0.722 | 12.512 | 0.130 | |
| **CHF with prior 30 days** | 1.336 | 0.914 | 1.953 | 0.135 | |
| **Acute Renal Failure (Pre-Op)** | 1.222 | 0.701 | 2.129 | 0.480 | |
| **Currently on Dialysis** | 1.446 | 1.086 | 1.925 | 0.012 | |
| **Disseminated Cancer** | 1.208 | 0.399 | 3.663 | 0.738 | |
| **Steroid use for chronic condition** | 1.549 | 1.143 | 2.101 | 0.005 | |
| **>10% loss body weight in last 6 months** | 1.588 | 0.875 | 2.883 | 0.129 | |
| **Preop Transfusion with 72 hrs prior to surgery** | 0.763 | 0.422 | 1.378 | 0.370 | |
| **Systemic Sepsis** |  |  |  |  | |
| None | 1.000 |  |  |  | |
| SIRS | 2.318 | 1.721 | 3.121 | <0.001 | |
| Sepsis | 3.985 | 2.474 | 6.419 | <0.001 | |
| Septic Shock | 12.714 | 2.728 | 59.245 | 0.001 | |
| **Hypertension requiring medications** | 0.968 | 0.765 | 1.224 | 0.785 | |
| **Dyspnea** |  |  |  |  | |
| No | 1.000 |  |  |  | |
| Moderate Exertion | 0.905 | 0.678 | 1.207 | 0.496 | |
| At Rest | 0.744 | 0.220 | 2.521 | 0.635 | |
| **Major Reintervention of Treated Segment** | | | | | |
|  | Odds Ratio | 95% lower bound | 95% upper bound | p-value | |
| **Procedure Type** |  |  |  |  | |
| ENDO | 1.000 |  |  |  | |
| OPEN-GSV | 1.456 | 1.082 | 1.958 | 0.013 | |
| OPEN-Other | 1.948 | 1.421 | 2.671 | <0.001 | |
| **Age** | 0.995 | 0.983 | 1.007 | 0.375 | |
| **Gender** |  |  |  |  | |
| female | 1.000 |  |  |  | |
| male | 0.991 | 0.769 | 1.276 | 0.941 | |
| **BMI** | 1.022 | 1.003 | 1.041 | 0.023 | |
| **Race** |  |  |  |  | |
| White | 1.000 |  |  |  | |
| Black or African American | 1.047 | 0.797 | 1.375 | 0.743 | |
| American Indian or Alaska Native | 1.000 |  |  |  | |
| Asian | 1.756 | 0.746 | 4.134 | 0.197 | |
| Native Hawaiian or Pacific Islander | 1.000 |  |  |  | |
| **High Risk Factors, Physiologic** | 0.976 | 0.706 | 1.350 | 0.885 | |
| **High Risk Factors, Anatomic** |  |  |  |  | |
| None | 1.000 |  |  |  | |
| Prior Bypass | 1.936 | 1.460 | 2.568 | <0.001 | |
| Prior Endovascular Intervention | 1.337 | 0.974 | 1.835 | 0.073 | |
| **Pre-procedural Antiplatelet Medication** | 0.863 | 0.628 | 1.187 | 0.365 | |
| **Pre-procedural Medication-Statin** | 1.080 | 0.816 | 1.430 | 0.589 | |
| **Diabetes** |  |  |  |  | |
| None | 1.000 |  |  |  | |
| Non-Insulin Dependent | 1.038 | 0.752 | 1.433 | 0.820 | |
| Insulin Dependent | 0.981 | 0.738 | 1.303 | 0.895 | |
| **Current Smoker within 1 year** | 0.988 | 0.750 | 1.300 | 0.930 | |
| **Acute Renal Failure (Pre-Op)** | 1.465 | 0.647 | 3.321 | 0.360 | |
| **Currently on Dialysis** | 1.077 | 0.683 | 1.700 | 0.750 | |
| **Open wound/wound infection** | 0.816 | 0.641 | 1.038 | 0.098 | |
| **Systemic Sepsis** |  |  |  |  | |
| None | 1.000 |  |  |  | |
| SIRS | 1.127 | 0.675 | 1.884 | 0.647 | |
| Sepsis | 0.710 | 0.220 | 2.289 | 0.566 | |
| Septic Shock | 3.746 | 0.436 | 32.193 | 0.229 | |
| **Hypertension requiring medications** | 0.923 | 0.671 | 1.270 | 0.621 | |
| **Major Adverse Cardiovascular Event** | | | | | |
|  | Odds Ratio | 95% lower bound | 95% upper bound | p-value | |
| **Procedure Type** |  |  |  |  | |
| ENDO | 1.000 |  |  |  | |
| OPEN-GSV | 2.987 | 1.849 | 4.827 | <0.001 | |
| OPEN-Other | 4.139 | 2.511 | 6.820 | <0.001 | |
| **Age** | 1.014 | 0.994 | 1.035 | 0.166 | |
| **Gender** |  |  |  |  | |
| female | 1.000 |  |  |  | |
| male | 0.781 | 0.538 | 1.134 | 0.195 | |
| **BMI** | 1.016 | 0.986 | 1.047 | 0.290 | |
| **Race** |  |  |  |  | |
| White | 1.000 |  |  |  | |
| Black or African American | 0.863 | 0.564 | 1.319 | 0.496 | |
| American Indian or Alaska Native | 1.918 | 0.245 | 14.999 | 0.535 | |
| Asian | 0.478 | 0.064 | 3.601 | 0.474 | |
| Native Hawaiian or Pacific Islander | 1.000 |  |  |  | |
| **High Risk Factors, Physiologic** | 1.115 | 0.693 | 1.794 | 0.654 | |
| **High Risk Factors, Anatomic** |  |  |  |  | |
| None | 1.000 |  |  |  | |
| Prior Bypass | 0.613 | 0.370 | 1.014 | 0.057 | |
| Prior Endovascular Intervention | 0.822 | 0.512 | 1.318 | 0.415 | |
| **Pre-procedural Antiplatelet Medication** | 1.666 | 0.907 | 3.063 | 0.100 | |
| **Pre-procedural Medication-Statin** | 1.153 | 0.738 | 1.802 | 0.532 | |
| **Diabetes** |  |  |  |  | |
| None | 1.000 |  |  |  | |
| Non-Insulin Dependent | 0.947 | 0.564 | 1.589 | 0.837 | |
| Insulin Dependent | 1.107 | 0.729 | 1.682 | 0.634 | |
| **Current Smoker within 1 year** | 0.753 | 0.469 | 1.206 | 0.238 | |
| **CHF with prior 30 days** | 0.938 | 0.441 | 1.996 | 0.868 | |
| **Currently on Dialysis** | 2.796 | 1.620 | 4.824 | <0.001 | |
| **Disseminated Cancer** | 3.260 | 1.214 | 8.754 | 0.019 | |
| **>10% loss body weight in last 6 months** |  |  |  |  | |
| **Systemic Sepsis** | 1.000 |  |  |  | |
| None | 1.677 | 0.900 | 3.125 | 0.104 | |
| SIRS | 1.000 |  |  |  | |
| Sepsis | 7.782 | 0.806 | 75.105 | 0.076 | |
| Septic Shock | 1.408 | 0.742 | 2.672 | 0.296 | |
| **Hypertension requiring medications** |  |  |  |  | |
| **Dyspnea** | 1.000 |  |  |  | |
| No | 2.159 | 1.360 | 3.428 | 0.001 | |
| Moderate Exertion | 1.514 | 0.195 | 11.767 | 0.692 | |
| At Rest | 1.264 | 0.820 | 1.948 | 0.290 | |
| **Pre-procedural Medication-Beta Blocker** | collinear |  |  |  | |
| **Bleeding Disorder** | 1.228 | 0.836 | 1.803 | 0.295 | |

**Supplemental Table S12:** Inverse-propensity weighted with regression adjustment analysis, Femoral-Tibial

| **ENDO vs. OPEN-GSV** | | | | |
| --- | --- | --- | --- | --- |
| **Variable** | **Risk Ratio** | **95% lower bound** | **95% upper bound** | **p-value** |
| Major Amputation | 1.17 | 0.85 | 1.60 | 0.33 |
| MALE | 1.06 | 0.84 | 1.33 | 0.63 |
| MALE or POD | 1.02 | 0.83 | 1.25 | 0.86 |
| POD | 0.74 | 0.48 | 1.13 | 0.16 |
| Major Reintervention | 0.87 | 0.63 | 1.19 | 0.38 |
| MACE | 0.33 | 0.20 | 0.54 | <0.001 |
| **ENDO vs. OPEN-Other** | | | | |
| **Variable** | **Risk Ratio** | **95% lower bound** | **95% upper bound** | **p-value** |
| Major Amputation | 0.79 | 0.57 | 1.11 | 0.18 |
| MALE | 0.81 | 0.63 | 1.03 | 0.09 |
| MALE or POD | 0.82 | 0.66 | 1.02 | 0.07 |
| POD | 0.78 | 0.47 | 1.27 | 0.31 |
| Major Reintervention | 0.68 | 0.48 | 0.95 | 0.025 |
| MACE | 0.27 | 0.16 | 0.45 | <0.001 |

**Supplemental Table S13:** Patient Demographics**,** Comparison of OPEN-GSV vs. ENDO vs. OPEN-Other, popliteal-tibial

|  | Procedure Type | | | | p-value | | |
| --- | --- | --- | --- | --- | --- | --- | --- |
|  | **OPEN-GSV**  **(n=1,037)** | **ENDO**  **(n=2,769)** | **OPEN-OTHER**  **(n=269)** | **Overall** | | **OPEN-GSV vs. ENDO** | **OPEN-Other vs. ENDO** |
| **Age** | 67.790 (12.190) | 69.384 (12.227) | 68.985 (10.126) | 0.001 | | <0.001 | 0.604 |
| **BMI** | 28.688 (6.107) | 28.405 (6.298) | 27.874 (5.792) | 0.146 | | 0.22 | 0.19 |
| **Female Sex** | 281 (27.1%) | 887 (32.0%) | 85 (31.6%) | 0.013 | | 0.003 | 0.884 |
| **RACE** |  |  |  |  | |  |  |
| American Indian or Alaska Native | 3 (0.3%) | 14 (0.6%) | 2 (0.9%) | 0.1 | | 0.16 | 0.15 |
| Asian | 13 (1.5%) | 63 (2.6%) | 6 (2.6%) |  | |  |  |
| Black or African American | 255 (29.3%) | 717 (29.6%) | 50 (22.0%) |  | |  |  |
| Native Hawaiian or Pacific Islander | 0 (0.0%) | 6 (0.2%) | 0 (0.0%) |  | |  |  |
| White | 598 (68.8%) | 1,620 (66.9%) | 169 (74.4%) |  | |  |  |
| **Functional Health Status** |  |  |  |  | |  |  |
| Independent | 924 (89.3%) | 2,277 (82.7%) | 242 (90.0%) | <0.001 | | <0.001 | 0.008 |
| Partially Dependent | 100 (9.7%) | 427 (15.5%) | 23 (8.6%) |  | |  |  |
| Totally Dependent | 11 (1.1%) | 48 (1.7%) | 4 (1.5%) |  | |  |  |
| **DIABETES** |  |  |  |  | |  |  |
| None | 365 (35.2%) | 890 (32.1%) | 104 (38.7%) | 0.018 | | 0.081 | 0.013 |
| Non-Insulin Dependent | 216 (20.8%) | 551 (19.9%) | 61 (22.7%) |  | |  |  |
| Insulin Dependent | 456 (44.0%) | 1,328 (48.0%) | 104 (38.7%) |  | |  |  |
| **Current Smoker within 1 year** | 258 (24.9%) | 457 (16.5%) | 63 (23.4%) | <0.001 | | <0.001 | 0.004 |
| **DYSPNEA** |  |  |  | 0.211 | | 0.2 | 0.206 |
| No | 944 (91.0%) | 2,479 (89.5%) | 250 (92.9%) |  | |  |  |
| Moderate Exertion | 90 (8.7%) | 271 (9.8%) | 18 (6.7%) |  | |  |  |
| At Rest | 3 (0.3%) | 19 (0.7%) | 1 (0.4%) |  | |  |  |
| **Ventilator Dependent** | 0 (0.0%) | 4 (0.1%) | 0 (0.0%) | 0.389 | | 0.221 | 0.533 |
| **History of Severe COPD** | 61 (5.9%) | 185 (6.7%) | 18 (6.7%) | 0.665 | | 0.372 | 0.995 |
| **Ascites** | 3 (0.3%) | 7 (0.3%) | 0 (0.0%) | 0.687 | | 0.845 | 0.409 |
| **CHF with prior 30 days** | 38 (3.7%) | 137 (4.9%) | 6 (2.2%) | 0.044 | | 0.092 | 0.045 |
| **Hypertension requiring medications** | 846 (81.6%) | 2,344 (84.7%) | 238 (88.5%) | 0.009 | | 0.022 | 0.094 |
| **Acute Renal Failure (Pre-Op)** | 23 (2.2%) | 61 (2.2%) | 6 (2.2%) | 0.999 | | 0.978 | 0.977 |
| **Currently on Dialysis** | 141 (13.6%) | 487 (17.6%) | 35 (13.0%) | 0.004 | | 0.003 | 0.057 |
| **Disseminated Cancer** | 6 (0.6%) | 11 (0.4%) | 0 (0.0%) | 0.406 | | 0.455 | 0.3 |
| **Open wound/wound infection** | 614 (59.2%) | 1,684 (60.8%) | 153 (56.9%) | 0.35 | | 0.367 | 0.207 |
| **Steroid use for chronic condition** | 69 (6.7%) | 231 (8.3%) | 19 (7.1%) | 0.2 | | 0.085 | 0.466 |
| **>10% loss body weight in last 6 months** | 11 (1.1%) | 48 (1.7%) | 2 (0.7%) | 0.18 | | 0.135 | 0.223 |
| **Bleeding Disorder** | 171 (19.3%) | 654 (29.6%) | 48 (21.3%) | <0.001 | | <0.001 | 0.009 |
| **Preop Transfusion with 72 hrs prior to surgery** | 31 (3.0%) | 48 (1.7%) | 4 (1.5%) | 0.041 | | 0.016 | 0.766 |
| **Systemic Sepsis** |  |  |  |  | |  |  |
| None | 975 (94.0%) | 2,499 (90.2%) | 255 (94.8%) | 0.005 | | 0.003 | 0.107 |
| SIRS | 41 (4.0%) | 192 (6.9%) | 10 (3.7%) |  | |  |  |
| Sepsis | 20 (1.9%) | 74 (2.7%) | 4 (1.5%) |  | |  |  |
| Septic Shock | 1 (0.1%) | 4 (0.1%) | 0 (0.0%) |  | |  |  |

**Supplemental Table S14:** Procedural Data, Comparison of OPEN-GSV vs. ENDO vs. OPEN-Other, popliteal-tibial

|  | Procedure Type | | | | p-value | | | |
| --- | --- | --- | --- | --- | --- | --- | --- | --- |
|  | **OPEN-GSV**  **(n=1,037)** | **ENDO**  **(n=2,769)** | **OPEN-OTHER**  **(n=269)** | **Overall** | | **OPEN-GSV vs. ENDO** | **OPEN-Other vs. ENDO** |  |
| **Symptomatology** |  |  |  |  | |  |  |  |
| Critical limb ischemia: rest pain | 237 (22.9%) | 494 (17.8%) | 89 (33.1%) | <0.001 | | <0.001 | <0.001 |  |
| Critical limb ischemia: tissue loss | 800 (77.1%) | 2,275 (82.2%) | 180 (66.9%) |  | |  |  |  |
| **High Risk Factors, Physiologic** | 316 (30.7%) | 1,131 (41.0%) | 76 (28.3%) | <0.001 | | <0.001 | <0.001 |  |
| **High Risk Factors, Anatomic** |  |  |  | <0.001 | | 0.007 | <0.001 |  |
| None | 721 (69.5%) | 2,007 (72.5%) | 126 (46.8%) |  | |  |  |  |
| Prior Bypass | 97 (9.4%) | 296 (10.7%) | 92 (34.2%) |  | |  |  |  |
| Prior Endovascular | 219 (21.1%) | 466 (16.8%) | 51 (19.0%) |  | |  |  |  |
| **Pre-procedural Antiplatelet Medication** | 803 (77.6%) | 2,210 (80.1%) | 229 (85.1%) | 0.018 | | 0.084 | 0.048 |  |
| **Pre-procedural Medication-Statin** | 722 (69.7%) | 1,926 (69.9%) | 198 (73.6%) | 0.428 | | 0.884 | 0.209 |  |
| **Pre-procedural Medication-Beta Blocker** | 578 (55.8%) | 1,708 (62.0%) | 181 (67.3%) | <0.001 | | <0.001 | 0.085 |  |
| **Overall Procedure** |  |  |  |  | |  |  |  |
| Angioplasty | - | 1,885 (68.1%) | - | <0.001 | | <0.001 | <0.001 |  |
| Atherectomy | - | 647 (23.4%) | - |  | |  |  |  |
| Bypass | 1,037 (100.0%) | - | 269 (100.0%) |  | |  |  |  |
| Stenting | - | 237 (8.6%) | - |  | |  |  |  |
| **ASACLAS** |  |  |  |  | |  |  |  |
| 1-No Disturb | 1 (0.1%) | 2 (0.1%) | 0 (0.0%) | 0.019 | | 0.022 | 0.083 |  |
| 2-Mild Disturb | 37 (3.6%) | 168 (6.4%) | 6 (2.2%) |  | |  |  |  |
| 3-Severe Disturb | 690 (66.7%) | 1,682 (63.9%) | 176 (65.4%) |  | |  |  |  |
| 4-Life Threat | 306 (29.6%) | 778 (29.5%) | 87 (32.3%) |  | |  |  |  |
| 5-Moribund | 1 (0.1%) | 3 (0.1%) | 0 (0.0%) |  | |  |  |  |
| **ANESTHES** |  |  |  |  | |  |  |  |
| None | 0 (0.0%) | 8 (0.3%) | 0 (0.0%) |  | |  |  |  |
| Epidural | 7 (0.7%) | 0 (0.0%) | 0 (0.0%) | <0.001 | | <0.001 | <0.001 |  |
| General | 956 (92.2%) | 880 (31.8%) | 266 (98.9%) |  | |  |  |  |
| Local | 0 (0.0%) | 23 (0.8%) | 0 (0.0%) |  | |  |  |  |
| MAC/IV Sedation | 10 (1.0%) | 1,818 (65.7%) | 1 (0.4%) |  | |  |  |  |
| Other | 1 (0.1%) | 6 (0.2%) | 0 (0.0%) |  | |  |  |  |
| Regional | 7 (0.7%) | 21 (0.8%) | 0 (0.0%) |  | |  |  |  |
| Spinal | 56 (5.4%) | 11 (0.4%) | 2 (0.7%) |  | |  |  |  |
| **Elective Surgery** | 484 (46.7%) | 1,256 (45.4%) | 127 (47.2%) | 0.688 | | 0.465 | 0.567 |  |
| **OPTIME** | 244.1 (102) | 106.9 (66.5) | 257.8 (124) | <0.001 | | <0.001 | <0.001 |  |

**Supplemental Table S15:** 30-day outcomes, Comparison of OPEN-GSV vs. ENDO vs. OPEN-Other, popliteal-tibial

|  | Procedure Type | | | p-value | | | |
| --- | --- | --- | --- | --- | --- | --- | --- |
|  | **OPEN-GSV**  **(n=1,037)** | **ENDO**  **(n=2,769)** | **OPEN-OTHER**  **(n=269)** | **Overall** | **OPEN-GSV vs. ENDO** | **OPEN-Other vs. ENDO** |  |
| **Total Length of Stay, in days** | 11.040 (9.0) | 6.772 (8.7) | 10.184 (9.1) | <0.001 | <0.001 | <0.001 |  |
| **Discharge Destination** |  |  |  |  |  |  |  |
| Against Medical Advice (AMA) | 1 (0.1%) | 7 (0.3%) | 0 (0.0%) | <0.001 | <0.001 | <0.001 |  |
| Expired | 18 (1.7%) | 27 (1.0%) | 6 (2.2%) |  |  |  |  |
| Home | 574 (55.6%) | 2,134 (77.3%) | 174 (64.9%) |  |  |  |  |
| Hospice | 3 (0.3%) | 11 (0.4%) | 0 (0.0%) |  |  |  |  |
| Rehab | 147 (14.2%) | 171 (6.2%) | 38 (14.2%) |  |  |  |  |
| Separate Acute Care | 9 (0.9%) | 31 (1.1%) | 1 (0.4%) |  |  |  |  |
| Skilled Care, Not Home | 278 (26.9%) | 377 (13.7%) | 46 (17.2%) |  |  |  |  |
| Unskilled Facility Not Home | 2 (0.2%) | 2 (0.1%) | 3 (1.1%) |  |  |  |  |
| **Non-Routine Discharge** | 445 (43.7%) | 608 (22.2%) | 89 (33.8%) | <0.001 | <0.001 | <0.001 |  |
| **Untreated Loss of Patency** | 23 (2.2%) | 42 (1.5%) | 7 (2.6%) | 0.193 | 0.137 | 0.177 |  |
| **Bleeding Requiring Transfusion or Secondary Procedure** | 213 (20.5%) | 203 (7.3%) | 70 (26.0%) | <0.001 | <0.001 | <0.001 |  |
| **Myocardial Infarction or Stroke** | 47 (4.5%) | 42 (1.5%) | 7 (2.6%) | <0.001 | <0.001 | 0.177 |  |
| **Wound Infection/Complication** | 130 (12.5%) | 60 (2.2%) | 38 (14.1%) | <0.001 | <0.001 | <0.001 |  |
| **Major Reintervention of Treated Segment** | 45 (4.3%) | 106 (3.8%) | 13 (4.8%) | 0.607 | 0.472 | 0.417 |  |
| **Major Amputation (Transtibial or Proximal** | 45 (4.3%) | 147 (5.3%) | 9 (3.3%) | 0.217 | 0.224 | 0.164 |  |
| **Death** | 30 (2.9%) | 76 (2.7%) | 11 (4.1%) | 0.451 | 0.804 | 0.207 |  |
| **Readmission** | 202 (22.1%) | 479 (20.8%) | 54 (23.0%) | 0.597 | 0.434 | 0.44 |  |
| **Unplanned Readmission** | 193 (21.3%) | 447 (19.7%) | 53 (22.6%) | 0.397 | 0.312 | 0.284 |  |
| **Related Readmission** | 136 (16.0%) | 244 (11.8%) | 39 (17.7%) | 0.001 | 0.002 | 0.011 |  |
| **Serious Complication** | 311 (30.0%) | 534 (19.3%) | 80 (29.7%) | <0.001 | <0.001 | <0.001 |  |
| **Any Complication** | 349 (33.7%) | 543 (19.6%) | 89 (33.1%) | <0.001 | <0.001 | <0.001 |  |
| **Cardiac Arrest** | 19 (1.8%) | 16 (0.6%) | 5 (1.9%) | <0.001 | <0.001 | 0.015 |  |
| **Myocardial Infarction** | 45 (4.3%) | 45 (1.6%) | 3 (1.1%) | <0.001 | <0.001 | 0.522 |  |
| **Cardiac Complication** | 60 (5.8%) | 57 (2.1%) | 7 (2.6%) | <0.001 | <0.001 | 0.553 |  |
| **Pneumonia** | 15 (1.4%) | 36 (1.3%) | 2 (0.7%) | 0.663 | 0.727 | 0.433 |  |
| **Deep Incisional SSI** | 23 (2.2%) | 6 (0.2%) | 3 (1.1%) | <0.001 | <0.001 | 0.01 |  |
| **Organ Space SSI** | 3 (0.3%) | 15 (0.5%) | 3 (1.1%) | 0.228 | 0.312 | 0.242 |  |
| **Superficial Incisional SSI** | 64 (6.2%) | 16 (0.6%) | 14 (5.2%) | <0.001 | <0.001 | <0.001 |  |
| **SSI** | 89 (8.6%) | 37 (1.3%) | 19 (7.1%) | <0.001 | <0.001 | <0.001 |  |
| **UTI** | 8 (0.8%) | 19 (0.7%) | 8 (3.0%) | <0.001 | 0.78 | <0.001 |  |
| **VTE** | 9 (0.9%) | 16 (0.6%) | 2 (0.7%) | 0.609 | 0.324 | 0.735 |  |
| **Acute Renal Failure** | 6 (0.6%) | 17 (0.6%) | 1 (0.4%) | 0.883 | 0.9 | 0.621 |  |
| **Progressive Renal Insufficiency** | 9 (0.9%) | 14 (0.5%) | 4 (1.5%) | 0.107 | 0.199 | 0.045 |  |
| **Renal Failure** | 14 (1.4%) | 30 (1.1%) | 5 (1.9%) | 0.473 | 0.493 | 0.255 |  |
| **Return to OR** | 220 (21.2%) | 388 (14.0%) | 58 (21.6%) | <0.001 | <0.001 | <0.001 |  |
| **Reoperation** | 220 (21.2%) | 388 (14.0%) | 58 (21.6%) | <0.001 | <0.001 | <0.001 |  |
| **Wound Distruption** | 15 (1.4%) | 2 (0.1%) | 6 (2.2%) | <0.001 | <0.001 | <0.001 |  |
| **DVT** | 7 (0.7%) | 12 (0.4%) | 2 (0.7%) | 0.562 | 0.346 | 0.473 |  |
| **PE** | 3 (0.3%) | 4 (0.1%) | 0 (0.0%) | 0.492 | 0.353 | 0.533 |  |
| **Unplanned Intubation** | 34 (3.3%) | 35 (1.3%) | 6 (2.2%) | <0.001 | <0.001 | 0.19 |  |
| **Failure to Wean** | 8 (0.8%) | 10 (0.4%) | 2 (0.7%) | 0.226 | 0.1 | 0.34 |  |
| **Stroke CVA** | 5 (0.5%) | 9 (0.3%) | 0 (0.0%) | 0.464 | 0.476 | 0.349 |  |
| **Septic Shock** | 12 (1.2%) | 23 (0.8%) | 0 (0.0%) | 0.179 | 0.347 | 0.133 |  |
| **Sepsis** | 22 (2.1%) | 72 (2.6%) | 7 (2.6%) | 0.693 | 0.397 | 0.998 |  |

**Supplemental Table S16:** Regression Analysis of 30-day outcomes, popliteal-tibial

| **Major Amputation (Transtibial or Proximal)** | | | | | |
| --- | --- | --- | --- | --- | --- |
|  | Odds Ratio | 95% lower bound | 95% upper bound | | p-value |
| Procedure Type |  |  |  | |  |
| ENDO | 1.000 |  |  | |  |
| OPEN-GSV | 0.768 | 0.510 | 1.157 | | 0.207 |
| OPEN-OTHER | 0.629 | 0.283 | 1.396 | | 0.255 |
| Age | 0.978 | 0.964 | 0.993 | | 0.004 |
| SEX |  |  |  | |  |
| female | 1.000 |  |  | |  |
| male | 1.242 | 0.859 | 1.797 | | 0.249 |
| BMI | 1.000 | 0.974 | 1.026 | | 0.995 |
| Race |  |  |  | |  |
| White | 1.000 |  |  | |  |
| Black or African American | 1.278 | 0.903 | 1.808 | | 0.166 |
| American Indian or Alaska Native | 1.088 | 0.140 | 8.464 | | 0.936 |
| Asian | 1.056 | 0.363 | 3.074 | | 0.921 |
| Native Hawaiian or Pacific Islander | 2.398 | 0.252 | 22.829 | | 0.447 |
| High Risk Factors, Physiologic | 1.013 | 0.658 | 1.561 | | 0.952 |
| High Risk Factors, Anatomic |  |  |  | |  |
| None | 1.000 |  |  | |  |
| Prior Bypass | 1.332 | 0.803 | 2.208 | | 0.267 |
| Prior ENDO | 0.999 | 0.636 | 1.569 | | 0.996 |
| Pre-procedural Antiplatelet Medication | 0.652 | 0.446 | 0.955 | | 0.028 |
| Pre-procedural Medication-Statin | 0.848 | 0.593 | 1.211 | | 0.363 |
| Functional Health Status |  |  |  | |  |
| Independent | 1.000 |  |  | |  |
| Partially Dependent | 1.221 | 0.770 | 1.937 | | 0.396 |
| Totally Dependent | 0.355 | 0.047 | 2.675 | | 0.315 |
| DIABETES |  |  |  | |  |
| None | 1.000 |  |  | |  |
| Non-Insulin Dependent | 0.882 | 0.552 | 1.408 | | 0.598 |
| Insulin Dependent | 0.714 | 0.484 | 1.053 | | 0.089 |
| Current Smoker within 1 year | 0.602 | 0.375 | 0.964 | | 0.035 |
| Ascites | 1.483 | 0.153 | 14.344 | | 0.734 |
| CHF with prior 30 days | 1.756 | 0.934 | 3.304 | | 0.081 |
| Acute Renal Failure (Pre-Op) | 0.823 | 0.309 | 2.193 | | 0.697 |
| Currently on Dialysis | 1.423 | 0.882 | 2.296 | | 0.148 |
| Disseminated Cancer | 2.017 | 0.251 | 16.194 | | 0.509 |
| Open wound/wound infection | 0.797 | 0.567 | 1.122 | | 0.193 |
| Steroid use for chronic condition | 1.361 | 0.800 | 2.315 | | 0.255 |
| >10% loss body weight in last 6 months | 1.896 | 0.728 | 4.936 | | 0.190 |
| Preop Transfusion with 72 hrs prior to surgery | 0.786 | 0.267 | 2.314 | | 0.662 |
| Systemic Sepsis |  |  |  | |  |
| None | 1.000 |  |  | |  |
| SIRS | 2.517 | 1.545 | 4.101 | | <0.001 |
| Sepsis | 3.496 | 1.720 | 7.105 | | 0.001 |
| Septic Shock | Collinear |  |  | |  |
| **Major Adverse Limb Event** | | | | | |
|  | Odds Ratio | 95% lower bound | 95% upper bound | p-value | |
| Procedure Type |  |  |  |  | |
| ENDO | 1.000 |  |  |  | |
| OPEN-GSV | 0.888 | 0.655 | 1.204 | 0.444 | |
| OPEN-OTHER | 0.866 | 0.513 | 1.460 | 0.588 | |
| Age | 0.979 | 0.968 | 0.991 | 0.001 | |
| SEX |  |  |  |  | |
| female | 1.000 |  |  |  | |
| male | 1.326 | 0.995 | 1.768 | 0.054 | |
| BMI | 1.002 | 0.981 | 1.023 | 0.863 | |
| Race |  |  |  |  | |
| White | 1.000 |  |  |  | |
| Black or African American | 1.061 | 0.803 | 1.403 | 0.675 | |
| American Indian or Alaska Native | 0.583 | 0.076 | 4.459 | 0.603 | |
| Asian | 1.586 | 0.782 | 3.214 | 0.201 | |
| Native Hawaiian or Pacific Islander | 1.732 | 0.187 | 16.014 | 0.628 | |
| High Risk Factors, Physiologic | 1.149 | 0.828 | 1.595 | 0.405 | |
| High Risk Factors, Anatomic |  |  |  |  | |
| None | 1.000 |  |  |  | |
| Prior Bypass | 1.761 | 1.232 | 2.517 | 0.002 | |
| Prior Endovascular | 1.069 | 0.758 | 1.508 | 0.704 | |
| Pre-procedural Antiplatelet Medication | 0.677 | 0.500 | 0.917 | 0.012 | |
| Pre-procedural Medication-Statin | 0.985 | 0.741 | 1.308 | 0.916 | |
| Functional Health Status |  |  |  |  | |
| Independent | 1.000 |  |  |  | |
| Partially Dependent | 1.204 | 0.833 | 1.739 | 0.323 | |
| Totally Dependent | 0.756 | 0.229 | 2.491 | 0.646 | |
| DIABETES |  |  |  |  | |
| None | 1.000 |  |  |  | |
| Non-Insulin Dependent | 0.928 | 0.651 | 1.323 | 0.679 | |
| Insulin Dependent | 0.720 | 0.530 | 0.979 | 0.036 | |
| Current Smoker within 1 year | 0.828 | 0.590 | 1.160 | 0.272 | |
| Ascites | 1.159 | 0.131 | 10.266 | 0.895 | |
| Acute Renal Failure (Pre-Op) | 1.223 | 0.577 | 2.592 | 0.600 | |
| Currently on Dialysis | 1.040 | 0.702 | 1.541 | 0.845 | |
| Disseminated Cancer | 1.038 | 0.130 | 8.312 | 0.972 | |
| Open wound/wound infection | 0.686 | 0.528 | 0.891 | 0.005 | |
| Steroid use for chronic condition | 1.399 | 0.922 | 2.121 | 0.114 | |
| >10% loss body weight in last 6 months | 1.429 | 0.604 | 3.379 | 0.416 | |
| Systemic Sepsis |  |  |  |  | |
| None | 1.000 |  |  |  | |
| SIRS | 2.057 | 1.356 | 3.121 | 0.001 | |
| Sepsis | 2.394 | 1.263 | 4.537 | 0.007 | |
| Septic Shock | 4.233 | 0.461 | 38.870 | 0.202 | |
| Hypertension requiring medications | 1.153 | 0.809 | 1.643 | 0.431 | |
| **Perioperative Death** | | | | | |
|  | Odds Ratio | 95% lower bound | 95% upper bound | p-value | |
| **Procedure Type** |  |  |  |  | |
| ENDO | 1.000 |  |  |  | |
| OPEN-GSV | 1.339 | 0.844 | 2.125 | 0.215 | |
| OPEN-Other | 2.446 | 1.223 | 4.891 | 0.011 | |
| **Age** | 1.034 | 1.012 | 1.056 | 0.002 | |
| **Gender** |  |  |  |  | |
| female | 1.000 |  |  |  | |
| male | 0.935 | 0.609 | 1.435 | 0.757 | |
| **BMI** | 0.980 | 0.948 | 1.014 | 0.254 | |
| **High Risk Factors, Physiologic** | 1.456 | 0.870 | 2.436 | 0.153 | |
| **High Risk Factors, Anatomic** |  |  |  |  | |
| None | 1.000 |  |  |  | |
| Prior Bypass | 0.598 | 0.263 | 1.362 | 0.221 | |
| Prior Endovascular Intervention | 1.378 | 0.866 | 2.194 | 0.176 | |
| **Pre-procedural Antiplatelet Medication** | 1.606 | 0.917 | 2.812 | 0.098 | |
| **Pre-procedural Medication-Statin** | 0.776 | 0.497 | 1.210 | 0.263 | |
| **Functional Health Status** |  |  |  |  | |
| Independent | 1.000 |  |  |  | |
| Partially Dependent | 1.709 | 1.053 | 2.774 | 0.030 | |
| Totally Dependent | 5.606 | 2.383 | 13.192 | <0.001 | |
| **Diabetes** |  |  |  |  | |
| None | 1.000 |  |  |  | |
| Non-Insulin Dependent | 0.909 | 0.510 | 1.620 | 0.747 | |
| Insulin Dependent | 0.980 | 0.609 | 1.578 | 0.934 | |
| **Current Smoker within 1 year** | 0.961 | 0.508 | 1.819 | 0.904 | |
| **Ascites** | 3.255 | 0.529 | 20.034 | 0.203 | |
| **CHF with prior 30 days** | 2.076 | 1.079 | 3.994 | 0.029 | |
| **Acute Renal Failure (Pre-Op)** | 1.464 | 0.623 | 3.441 | 0.382 | |
| **Currently on Dialysis** | 2.868 | 1.729 | 4.757 | <0.001 | |
| **Disseminated Cancer** | 3.459 | 0.429 | 27.869 | 0.244 | |
| **Open wound/wound infection** | 1.006 | 0.657 | 1.539 | 0.979 | |
| **Steroid use for chronic condition** | 1.340 | 0.668 | 2.688 | 0.410 | |
| **>10% loss body weight in last 6 months** | 2.350 | 0.882 | 6.265 | 0.088 | |
| **Preop Transfusion with 72 hrs prior to surgery** | 1.312 | 0.480 | 3.585 | 0.596 | |
| **Systemic Sepsis** |  |  |  |  | |
| None | 1.000 |  |  |  | |
| SIRS | 2.075 | 1.114 | 3.866 | 0.021 | |
| Sepsis | 5.009 | 2.353 | 10.664 | <0.001 | |
| Septic Shock | 10.079 | 0.911 | 111.563 | 0.060 | |
| **Hypertension requiring medications** | 0.723 | 0.422 | 1.240 | 0.239 | |
| **Dyspnea** |  |  |  |  | |
| No | 1.000 |  |  |  | |
| Moderate Exertion | 1.556 | 0.868 | 2.790 | 0.137 | |
| At Rest | 0.837 | 0.102 | 6.893 | 0.869 | |
| **Pre-procedural Medication-Beta Blocker** | 1.024 | 0.656 | 1.599 | 0.916 | |
| **History of Severe COPD** | 0.609 | 0.251 | 1.479 | 0.273 | |
| **MALE or Perioperative Death** | | | | | |
|  | Odds Ratio | 95% lower bound | 95% upper bound | p-value | |
| **Procedure Type** |  |  |  |  | |
| ENDO | 1.000 |  |  |  | |
| OPEN-GSV | 1.016 | 0.780 | 1.323 | 0.907 | |
| OPEN-Other | 1.123 | 0.723 | 1.745 | 0.606 | |
| **Age** | 0.990 | 0.980 | 1.001 | 0.076 | |
| **Gender** |  |  |  |  | |
| female | 1.000 |  |  |  | |
| male | 1.120 | 0.875 | 1.435 | 0.368 | |
| **BMI** | 0.993 | 0.975 | 1.012 | 0.464 | |
| **Race** |  |  |  |  | |
| White | 1.000 |  |  |  | |
| Black or African American | 0.965 | 0.753 | 1.238 | 0.781 | |
| American Indian or Alaska Native | 0.433 | 0.057 | 3.292 | 0.419 | |
| Asian | 1.104 | 0.548 | 2.222 | 0.782 | |
| Native Hawaiian or Pacific Islander | 1.202 | 0.134 | 10.753 | 0.869 | |
| **High Risk Factors, Physiologic** | 1.227 | 0.920 | 1.636 | 0.164 | |
| **High Risk Factors, Anatomic** |  |  |  |  | |
| None | 1.000 |  |  |  | |
| Prior Bypass | 1.544 | 1.107 | 2.152 | 0.010 | |
| Prior Endovascular Intervention | 1.140 | 0.851 | 1.528 | 0.380 | |
| **Pre-procedural Antiplatelet Medication** | 0.789 | 0.599 | 1.040 | 0.093 | |
| **Pre-procedural Medication-Statin** | 0.931 | 0.725 | 1.195 | 0.574 | |
| **Functional Health Status** |  |  |  |  | |
| Independent | 1.000 |  |  |  | |
| Partially Dependent | 1.261 | 0.923 | 1.724 | 0.145 | |
| Totally Dependent | 1.942 | 0.938 | 4.019 | 0.074 | |
| **Diabetes** |  |  |  |  | |
| None | 1.000 |  |  |  | |
| Non-Insulin Dependent | 0.892 | 0.651 | 1.222 | 0.476 | |
| Insulin Dependent | 0.741 | 0.568 | 0.966 | 0.027 | |
| **Current Smoker within 1 year** | 0.854 | 0.626 | 1.164 | 0.317 | |
| **Ascites** | 2.583 | 0.579 | 11.521 | 0.214 | |
| **CHF with prior 30 days** | 1.503 | 0.934 | 2.418 | 0.093 | |
| **Acute Renal Failure (Pre-Op)** | 1.451 | 0.794 | 2.651 | 0.226 | |
| **Currently on Dialysis** | 1.344 | 0.966 | 1.870 | 0.079 | |
| **Disseminated Cancer** | 1.801 | 0.382 | 8.484 | 0.457 | |
| **Steroid use for chronic condition** | 1.345 | 0.922 | 1.963 | 0.124 | |
| **>10% loss body weight in last 6 months** | 1.421 | 0.669 | 3.018 | 0.360 | |
| **Preop Transfusion with 72 hrs prior to surgery** | 1.213 | 0.625 | 2.356 | 0.568 | |
| **Systemic Sepsis** |  |  |  |  | |
| None | 1.000 |  |  |  | |
| SIRS | 2.078 | 1.433 | 3.015 | <0.001 | |
| Sepsis | 2.796 | 1.633 | 4.786 | <0.001 | |
| Septic Shock | 6.224 | 0.996 | 38.872 | 0.050 | |
| **Hypertension requiring medications** | 0.980 | 0.723 | 1.328 | 0.896 | |
| **Dyspnea** |  |  |  |  | |
| No | 1.000 |  |  |  | |
| Moderate Exertion | 0.873 | 0.588 | 1.298 | 0.503 | |
| At Rest | 0.687 | 0.152 | 3.105 | 0.626 | |
| **Major Reintervention of Treated Segment** | | | | | |
|  | Odds Ratio | 95% lower bound | 95% upper bound | p-value | |
| **Procedure Type** |  |  |  |  | |
| ENDO | 1.000 |  |  |  | |
| OPEN-GSV | 1.166 | 0.786 | 1.730 | 0.446 | |
| OPEN-Other | 1.054 | 0.555 | 2.002 | 0.872 | |
| **Age** | 0.988 | 0.971 | 1.005 | 0.157 | |
| **Gender** |  |  |  |  | |
| female | 1.000 |  |  |  | |
| male | 1.496 | 0.996 | 2.248 | 0.053 | |
| **BMI** | 1.000 | 0.971 | 1.029 | 0.976 | |
| **Race** |  |  |  |  | |
| White | 1.000 |  |  |  | |
| Black or African American | 0.903 | 0.605 | 1.346 | 0.616 | |
| American Indian or Alaska Native | 1.000 |  |  |  | |
| Asian | 2.256 | 0.984 | 5.173 | 0.055 | |
| Native Hawaiian or Pacific Islander | 1.000 |  |  |  | |
| **High Risk Factors, Physiologic** | 1.062 | 0.672 | 1.678 | 0.797 | |
| **High Risk Factors, Anatomic** |  |  |  |  | |
| None | 1.000 |  |  |  | |
| Prior Bypass | 2.384 | 1.535 | 3.703 | <0.001 | |
| Prior Endovascular Intervention | 1.188 | 0.744 | 1.897 | 0.470 | |
| **Pre-procedural Antiplatelet Medication** | 0.778 | 0.506 | 1.198 | 0.254 | |
| **Pre-procedural Medication-Statin** | 1.100 | 0.738 | 1.640 | 0.639 | |
| **Diabetes** |  |  |  |  | |
| None | 1.000 |  |  |  | |
| Non-Insulin Dependent | 0.923 | 0.581 | 1.465 | 0.733 | |
| Insulin Dependent | 0.603 | 0.393 | 0.925 | 0.020 | |
| **Current Smoker within 1 year** | 1.037 | 0.668 | 1.609 | 0.872 | |
| **Acute Renal Failure (Pre-Op)** | 1.650 | 0.563 | 4.839 | 0.361 | |
| **Currently on Dialysis** | 0.832 | 0.459 | 1.508 | 0.544 | |
| **Open wound/wound infection** | 0.629 | 0.440 | 0.900 | 0.011 | |
| **Systemic Sepsis** |  |  |  |  | |
| None | 1.000 |  |  |  | |
| SIRS | 1.196 | 0.589 | 2.428 | 0.621 | |
| Sepsis | 1.069 | 0.322 | 3.549 | 0.913 | |
| Septic Shock | 10.055 | 1.064 | 95.004 | 0.044 | |
| **Hypertension requiring medications** | 1.328 | 0.804 | 2.195 | 0.268 | |
| **Major Adverse Cardiovascular Event** | | | | | |
|  | Odds Ratio | 95% lower bound | 95% upper bound | p-value | |
| **Procedure Type** |  |  |  |  | |
| ENDO | 1.000 |  |  |  | |
| OPEN-GSV | 3.709 | 2.144 | 6.418 | <0.001 | |
| OPEN-Other | 2.623 | 1.024 | 6.721 | 0.045 | |
| **Age** | 1.008 | 0.983 | 1.035 | 0.518 | |
| **Gender** |  |  |  |  | |
| female | 1.000 |  |  |  | |
| male | 0.814 | 0.466 | 1.422 | 0.470 | |
| **BMI** | 0.976 | 0.933 | 1.021 | 0.291 | |
| **Race** |  |  |  |  | |
| White | 1.000 |  |  |  | |
| Black or African American | 0.730 | 0.398 | 1.338 | 0.308 | |
| American Indian or Alaska Native | 1.000 |  |  |  | |
| Asian | 1.777 | 0.480 | 6.581 | 0.389 | |
| Native Hawaiian or Pacific Islander | 1.000 |  |  |  | |
| **High Risk Factors, Physiologic** | 1.527 | 0.793 | 2.940 | 0.205 | |
| **High Risk Factors, Anatomic** |  |  |  |  | |
| None | 1.000 |  |  |  | |
| Prior Bypass | 1.080 | 0.458 | 2.546 | 0.860 | |
| Prior Endovascular Intervention | 1.611 | 0.905 | 2.866 | 0.105 | |
| **Pre-procedural Antiplatelet Medication** | 0.892 | 0.450 | 1.770 | 0.744 | |
| **Pre-procedural Medication-Statin** | 0.980 | 0.530 | 1.813 | 0.950 | |
| **Diabetes** |  |  |  |  | |
| None | 1.000 |  |  |  | |
| Non-Insulin Dependent | 2.246 | 1.002 | 5.034 | 0.049 | |
| Insulin Dependent | 2.841 | 1.395 | 5.788 | 0.004 | |
| **Current Smoker within 1 year** | 0.955 | 0.452 | 2.021 | 0.905 | |
| **CHF with prior 30 days** | 1.401 | 0.503 | 3.907 | 0.519 | |
| **Currently on Dialysis** | 1.484 | 0.726 | 3.035 | 0.279 | |
| **Disseminated Cancer** | 2.727 | 0.704 | 10.563 | 0.146 | |
| **>10% loss body weight in last 6 months** |  |  |  |  | |
| **Systemic Sepsis** | 1.000 |  |  |  | |
| None | 2.954 | 1.379 | 6.329 | 0.005 | |
| SIRS | 0.601 | 0.080 | 4.540 | 0.622 | |
| Sepsis | 1.000 |  |  |  | |
| Septic Shock | 1.176 | 0.496 | 2.786 | 0.713 | |
| **Hypertension requiring medications** |  |  |  |  | |
| **Dyspnea** | 1.000 |  |  |  | |
| No | 1.384 | 0.634 | 3.024 | 0.414 | |
| Moderate Exertion | 1.000 |  |  |  | |
| At Rest | 1.705 | 0.898 | 3.240 | 0.103 | |
| **Pre-procedural Medication-Beta Blocker** | Collinear |  |  |  | |
| **Bleeding Disorder** | 1.785 | 1.044 | 3.052 | 0.034 | |

**Supplemental Table S17:** Inverse-propensity weighted with regression adjustment analysis, popliteal-tibial

| **ENDO vs. OPEN-GSV** | | | | |
| --- | --- | --- | --- | --- |
| **Variable** | **Risk Ratio** | **95% lower bound** | **95% upper bound** | **p-value** |
| Major Amputation | 1.21 | 0.84 | 1.76 | 0.31 |
| MALE | 1.16 | 0.88 | 1.54 | 0.30 |
| MALE or POD | 1.04 | 0.82 | 1.31 | 0.75 |
| POD | 0.82 | 0.52 | 1.29 | 0.38 |
| Major Reintervention | 0.89 | 0.61 | 1.31 | 0.55 |
| MACE | 0.31 | 0.20 | 0.49 | <0.001 |
| **ENDO vs. OPEN-Other** | | | | |
| **Variable** | **Risk Ratio** | **95% lower bound** | **95% upper bound** | **p-value** |
| Major Amputation | 1.27 | 0.62 | 2.62 | 0.51 |
| MALE | 1.00 | 0.60 | 1.66 | 1.00 |
| MALE or POD | 0.79 | 0.54 | 1.15 | 0.22 |
| POD | 0.56 | 0.33 | 0.96 | 0.036 |
| Major Reintervention | 0.63 | 0.33 | 1.21 | 0.17 |
| MACE | 0.44 | 0.18 | 1.03 | 0.058 |
